# Morphological and Physiological Indicators and Transcriptome Analyses Reveal the Mechanism of Selenium Multilevel Mitigation of Cadmium Damage in *Brassica juncea*

**DOI:** 10.3390/plants12081583

**Published:** 2023-04-07

**Authors:** Linling Li, Shiyan Wang, Shuai Wu, Shen Rao, Li Li, Shuiyuan Cheng, Hua Cheng

**Affiliations:** 1 School of Modern Industry for Selenium Science and Engineering, Wuhan Polytechnic University, Wuhan 430048, China; 12622@whpu.edu.cn (L.L.); harry1437@126.com (S.W.);; 2 National R&D Center for Se-rich Agricultural Products Processing, Wuhan Polytechnic University, Wuhan 430023, China

**Keywords:** selenomethionine, selenocysteine, cell wall, pectin, bivalent cation transporter

## Abstract

Cadmium (Cd) is a common agricultural soil pollutant, which does serious harm to the environment and the human body. In this study, *Brassica juncea* was treated with different concentrations of CdCl_2_ and Na_2_SeO_3_. Then, physiological indexes and transcriptome were measured to reveal the mechanisms by which Se reduces the inhibition and toxicity of Cd in *B. juncea*. The results showed that Se alleviated the inhibitive Cd effects on seedling biomass, root length, and chlorophyll, and promoted the adsorption of Cd by pectin and lignin in the root cell wall (CW). Se also alleviated the oxidative stress induced by Cd, and reduced the content of MDA in cells. As a result, SeCys and SeMet alleviated the transport of Cd to the shoots. Transcriptome data showed that the bivalent cation transporter MPP and ABCC subfamily participated in the separation of Cd in vacuoles, CAL1 was related to the chelation of Cd in the cytoplasm of cells, and ZIP transporter 4 reduced the transport of Cd to the shoots. These results indicated that Se alleviated the damage of Cd in plants and decreased its transport to the shoots by improving the antioxidant system, enhancing the ability of the CW to adsorb Cd, reducing the activity of Cd transporters, and chelating Cd.

## 1. Introduction

Heavy metals are a prominent category of environmental contaminants that pose substantial health risks to humans through the intake of heavy metal-containing foods, particularly cereals. Cd is a common harmful agricultural soil pollutant caused by various human activities, including electroplating, mining, smelting, and the use of pesticides [1]. The distribution of Cd in the soil causes oxidative damage to plants and produces toxic effects in crops. Under Cd stress, plants display a variety of toxic symptoms, such as chlorosis, disturbed ion homeostasis, photosynthetic suppression, disturbed metabolism, and growth suppression [2,3]. It is known that Cd alters photosynthesis and plant biomass and decreases the uptake of essential nutrients from the soil, ultimately altering plant growth [4]. Cd induces the production of reactive oxygen species (ROS), which directly or indirectly impairs the structures and functions of biological macromolecules. The massive production of ROS can lead to damage and dysfunction of DNA, genes, proteins, membranes, etc. In serious cases, such events can lead to cell death [5]. The damage to organelle structure and ultrastructure is also a common phenomenon of Cd toxicity. Furthermore, accumulated-crop Cd enters the food chain, posing a potential health risk to animals and humans [6]. Therefore, Cd is the greatest threat to plant growth and human health [3].

Cd usually exists in the soil in the form of Cd^2+^, which is considered the form of Cd with the highest toxicity. The Cd content of plant shoots is mostly a function of its absorption through roots, vacuole retention, xylem isolation, and phloem load [7]. For plants growing in Cd-containing soil, the Cd content of the aboveground part is generally lower than that of the root tissue. The following are considered the four main steps in Cd uptake and its movement to the seeds, i.e., root absorption, xylem loading, transfer to shoots, and, finally, the phloem transfer to the seeds [7,8]. Roots are the first plant part to take in Cd and other nutrients from the soil. Currently, multiple heavy metal transmembrane transporters have been described in various plants. Reports have suggested that Fe-transporting channels (e.g., OsNRAMP1) were also involved in Cd absorption and transport [9]. Another critical group of metal transporters is heavy metal ATPases (HMAs), which were found in multiple plant species. In rice, OsHMA3 mediates Cd sequestration in root vacuoles, thus decreasing Cd transport to the aboveground parts [10]. In a recent report, Cai demonstrated the overexpression of OsHMA3 in tobacco reduced by Cd in the shoots and improved the tolerance of tobacco to Cd [11]. OsHMA3 was found in tonoplasts, while OsHMA2 was active in the cell membrane, where it mediated the transport of Cd and Zn [12].

Se is one of the essential trace elements in animals and humans. Although not required for plants, Se has beneficial and important functions in many biological systems of plants [13,14]. In recent years, Se has attracted extensive attention in alleviating the toxicity of heavy metals in various plants and was considered a very promising element in plant resistance to heavy-metals-related stress [3,7,15]. Accordingly, Se reduces plant damage by Cd through many mechanisms: (1) it reduces the accessibility of soil Cd [16]; (2) it regulates the plant’s antioxidant system to reduce ROS-induced damage [3,17]; (3) it limits the subcellular distribution of Cd [18]; and (4) it forms heavy metal phytochelatins and aids vacuolar sequestration [19]. In addition, studies have suggested that Se downregulates the genes related to Cd transport, promoting the biosynthesis of lignin, and thus reducing the bioavailability of Cd, which was also a mechanism for the detoxification of Cd [20].

*B. juncea*, a Brassicaceae, represents a top oilseed crop worldwide [21]. *B. juncea* can highly absorb and accumulate Cd but has limited geographical distribution [22]. *B. juncea* was often used as a research material for heavy metal pollution. In the present study, *B. juncea* was treated with different concentrations of CdCl_2_ and Na_2_SeO_3_. Physiological indexes, morphology, and the Cd and Se contents in shoots and roots of *B. juncea* were measured and assessed as well as the changes in Se morphology. We further sequenced the transcriptome of different samples after treatment, combined with the changes in the forms and contents of Cd and Se, and analyzed genes that may be involved in Cd stress to explore the mechanism of Se in alleviating Cd stress and injury in *B. juncea*.

## 2. Results

### 2.1. Effects of Cd and Se on Seedling Growth Parameters

**Figure 1:**
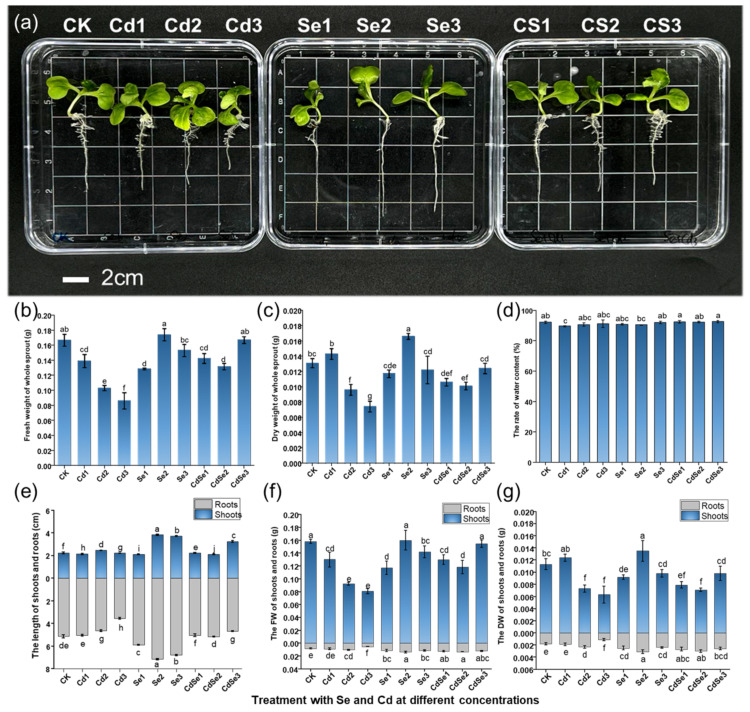
The effects of Na_2_SeO_3_ and CdCl_2_ treatment on the growth of *B. juncea* seedlings. The white bar represents the 2 cm length in the picture. Five seedlings were randomly selected for parameter measurements and the average values were taken. The measurements were repeated three times for each treatment. The error bar represents the standard deviation in the histogram. The lowercase letter a to j on the bar reflects the 5% significance level. (**a**) Growth morphology of *B. juncea* seedlings under different treatment conditions; (**b**) the fresh weight of whole seedlings; (**c**) the dry weight of whole seedlings; (**d**) the percentage of seedling water content; (**e**) the root and shoot lengths of seedlings under different treatments; (**f**) the fresh weight of seedling roots and shoots; and (**g**) the dry weight of seedling roots and shoots.

Se and Cd both affect the growth and development of seedlings. Cd treatment inhibited the growth of the shoots and roots of seedlings, while the addition of Se alleviated the inhibitive effects of Cd on seedling growth (Figure 1a). With the increase in Cd concentration, the shoots of seedlings increased first and then decreased, while the roots became shorter with increasing Cd concentration. Compared to the control, Se treatment significantly increased the growth of shoots and roots. When the treatment concentration was Se2, the shooting part reached the highest height, about 3.82 cm, and the root also reached 7.15 cm. When the seedlings were treated with Se and Cd at the same time, the roots became longer with increasing Se concentration, while the length of the shoot reached 3.24 cm when the Se concentration was Se3, which was 0.79 cm longer than that of Cd alone, with a statistically significant difference (Figure 1e).

Cd treatment significantly reduced the fresh (FW) and dry weight (DW) of seedlings (Figure 1b,c). With increasing Cd concentration, the weight of the whole seedling decreased significantly, and the FW decreased to only 0.0594 g at the Cd concentration of Cd3. However, the FW and DW of seedlings increased first and then decreased under Se treatment alone. When seedlings were treated with Se and Cd, with the increasing Se concentration, the FW and DW of seedlings decreased first and then increased. At the Cd concentration of CdSe3, the FW returned to the control level, while the DW was slightly lower than the control level. In the root parts of seedlings, Se treatment effectively alleviated the effect of Cd on the roots of seedlings, and both FW and DW were higher than with Cd treatment alone (Figure 1f,g).

### 2.2. Effects of Cd and Se on Root Tips of Seedlings

**Figure 2:**
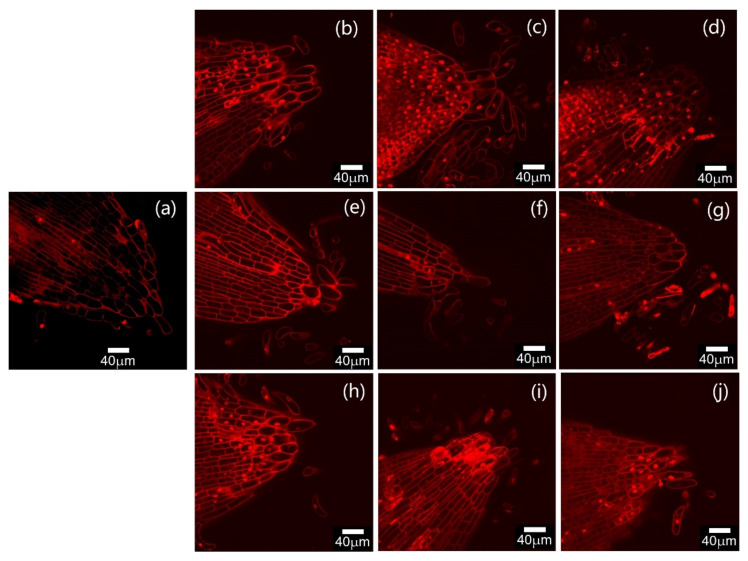
Laser scanning microscopy (LSM) imaging of root tip viability by propidium iodide (PI) staining, where the red fluorescence indicates dead cells. The bar represents a length of 40 μm. (**a**) Control group; (**b**) Cd1—treatment of seedling root tips with 10 mg/L CdCl_2_; (**c**) Cd2—50 mg/L CdCl_2_; (**d**) Cd3—100 mg/L CdCl_2_; (**e**) Se1 treatment of seedling root tips with 10 mg/L Na_2_SeO_3_; (**f**) Se2—50 mg/L Na_2_SeO_3_; (**g**) Se3—100 mg/L Na_2_SeO_3_; (**h**) CdSe1—50 mg/L CdCl_2_ and 10 mg/L Na_2_SeO_3_; (**i**) CdSe2—50 mg/L CdCl_2_ and 50 mg/L Na_2_SeO_3_; and (**j**) CdSe3—50 mg/L CdCl_2_ and 100 mg/L Na_2_SeO_3_.

PI (propidium iodide) staining can show the degree of damage to the root tip under a laser confocal microscope. Figure 2a shows that the root tip of the seedling was only lightly stained in the CW gap. The root tip nuclei of seedlings cultured in the medium with a low concentration of Cd (Cd 1) were stained (Figure 2b). With the increase in Cd concentration, the cytoplasm and nucleus were stained more deeply, and the stained root tip cells occupied most of the cells (Figure 2c,d). In addition, the root tips of the seedlings became thicker after Cd treatment. The root tips of seedlings treated with Se alone were less damaged, and only a few nuclei of cells in the root tips were stained at a high Se concentration (Se3) (Figure 2e–g). In the Se and Cd co-treatment group, the root tip cell damage was similar to that of the Cd1 treatment when the Se concentration was low (CdSe1) (Figure 2h), but weaker than that of the Cd2 treatment. When the concentration of Se increased to the levels in CdSe2, the nuclear staining of the root tips of seedlings decreased significantly (Figure 2i). When the concentration of Se increased to the levels in CdSe3, the staining of root tip nuclei increased, but was lower than that at the Cd2 treatment level (Figure 2j). The results indicated that the Se concentrations in Se2 and Se3 could alleviate the damage of Cd to the root tips of seedlings.

The microscope observation results showed that Cd caused greater damage to the root tips of seedlings when compared to the control. When the Cd concentration was that of Cd2, the root tip began to shrink. When the Cd concentration was that of Cd3, the root tip was necrotic. Se treatment had almost no effect on the root tips of plants. When the Se concentration was that of Se1, the growth state of the root tip was basically the same as that of the control. When the Se treatment concentration was that of Se2, the root tip staining deepens, but the root tip was not damaged or contracted; when the Se concentration was that of Se3, the root tip shrunk; the combined treatment of Se and Cd could alleviate the Cd damage caused to the root tip of the seedling, and the root hairs were significantly increased (Figure S1).

### 2.3. Effects of Cd and Se on the Photosynthesis of Seedlings

**Figure 3:**
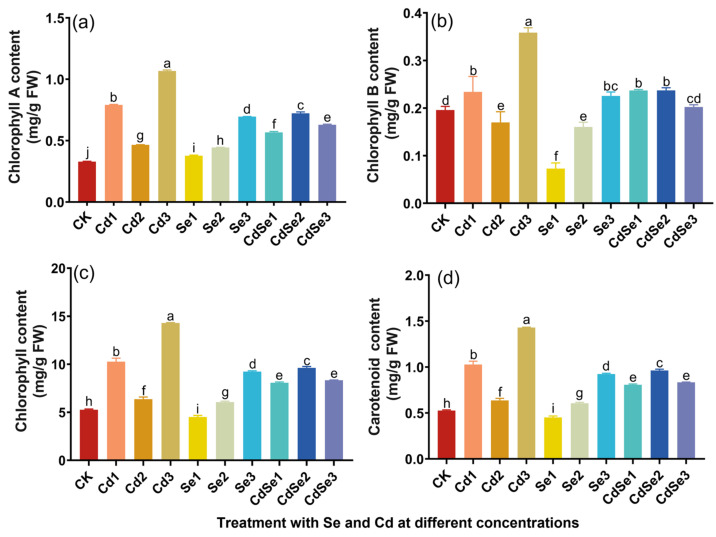
Changes in photosynthetic pigment content in mustard with different concentrations of Se and Cd. The error bar represents the standard deviation. The lowercase letter a to j on the bar reflects the 5% significance level. (**a**) Chlorophyll A content in seedling leaves; (**b**) chlorophyll B content in seedling leaves; (**c**) total chlorophyll content in seedling leaves; and (**d**) carotenoid content in seedling leaves.

The Cd treatment had a great effect on the chlorophyll content of seedlings (Figure 3). In the low-concentration Cd1 treatment, the total chlorophyll level, chlorophyll A (Figure 3a), and chlorophyll B (Figure 3b) were higher than the control. The chlorophyll level decreased significantly when the Cd concentration was higher (Cd2). When the Cd concentration reached Cd3, the chlorophyll level increased again, becoming significantly higher than in other treatment groups. The chlorophyll A level of the Se treatment group was higher than that of the control group (Figure 3a). With the increase in treatment concentration, the chlorophyll content gradually increased. The total chlorophyll level of Se2 and Se3 was higher than that of the control. When Cd and Se were applied to seedlings at the same time, the total chlorophyll level of CdSe1 was higher than that of the control and of Cd2 when the Se concentration was low. With the increase in Se concentration to CdSe2 levels, the total chlorophyll level also increased to 1.5 times that of Cd2 (Figure 3c). This indicated that Se treatment effectively alleviated Cd damage caused to the photosynthetic pigments.

In plant photosynthesis, carotenoids are auxiliary pigments in the light absorption process. Carotenoids can prevent the formation of singlet oxygen free radicals and protect plants from oxygen damage. The carotenoid content of seedlings treated with Cd alone was generally high. When the Cd concentration was at Cd3 levels, the carotenoid content was about 2.5 times that of the control. When only Se treatment was applied to the seedlings, the carotenoid content gradually increased with increasing treatment concentrations and reached 1.8 times the control at the Se concentration of Se3. When the seedlings were treated with both Se and Cd, the carotenoid content first increased and then decreased, and was higher than that of Cd2 alone, on the whole (Figure 3d).

### 2.4. Effects of Cd and Se on the Antioxidant System of Seedlings

**Figure 4:**
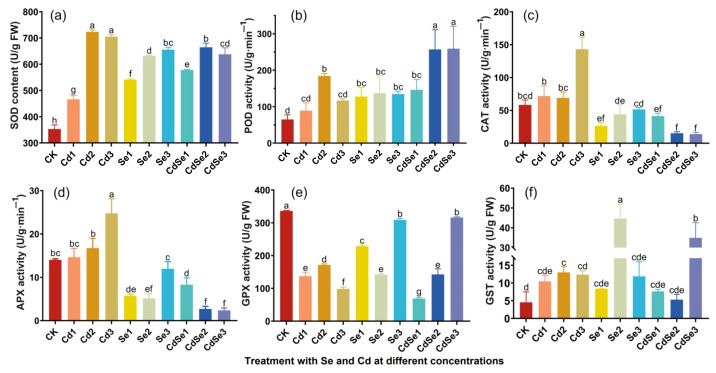
Changes in antioxidant enzyme activity of *B. juncea* seedlings under different concentrations of Se and Cd. The error bar represents the standard deviation. The lowercase letter a to h on the bar reflects the 5% significance level. (**a**) Superoxide dismutase activity; (**b**) peroxidase activity; (**c**) catalase activity; (**d**) ascorbate peroxidase activity; (**e**) glutathione peroxidase activity; and (**f**) glutathione transferase activity.

Cd and Se treatments had significant effects on the SOD activity of seedlings (Figure 4a). When the Cd concentrations were at Cd2 and Cd3 levels, the activity of SOD was significantly higher than that of other treatment groups. The activity of SOD in the Cd2 treatment group was the highest, followed by that in the Cd3 treatment group. However, when Cd and Se were treated together, the SOD activity was significantly lower than that of Cd2 alone, among which, the CdSe1 treatment was the lowest, followed by CdSe2 and CdSe3. The results showed that the SOD in seedlings positively responded to Cd-induced oxygen stress under Cd treatment, while Se treatment decreased the accumulation of active oxygen under Cd stress.

Among all treatments, CdSe2 and CdSe3 had the highest POD activity, followed by Cd2. Compared with Cd treatment alone, CdSe1 treatment significantly reduced the activity of POD (Figure 4b). The results indicated that a high concentration of Se did not significantly reduce POD activity.

In the determination of CAT activity, the Cd3 treatment had the highest CAT activity, followed by Cd1 and Cd2 treatments (Figure 4c). However, after the treatment of Cd and Se, the CAT activity decreased significantly. Among the treatments of CdSe2 and CdSe3, the enzyme activity of CAT was the lowest. It indicated that a higher concentration of Se was beneficial in reducing the level of hydrogen peroxide in seedlings, and the peroxide caused by Cd stress was mainly eliminated by CAT.

The Cd3 treatment had the greatest effect on the APX enzyme activity of seedlings, while the Se treatment could significantly reduce APX enzyme activity, among which CdSe2 and CdSe3 treatments had the lowest APX enzyme activity. Similarly, Se alleviated the effect of Cd on plant APX enzyme activity (Figure 4d).

When Cd was treated alone, the GPX activity of seedlings was significantly lower than that of the control (Figure 4e). The GPX enzyme activity of seedlings treated with Se3 and CdSe3 was slightly lower than that of the control, but significantly higher than that of other treatments. The activity of the GPX enzyme in the CdSe1 treatment was lower than that in the Cd2 treatment alone, which was at the lowest level. It indicated that a high concentration of Se effectively enhanced GPX enzyme activity under Cd stress.

The activity of GST enzyme in seedlings was significantly increased regardless of whether Se was treated alone or with Cd (Figure 4f). Among them, when the Se concentration was at the level of Se2, the activity of the GST enzyme was the highest, followed by CdSe3-treated seedlings. It indicated that the addition of Se effectively enhanced the GST enzyme activity of seedlings.

**Figure 5:**
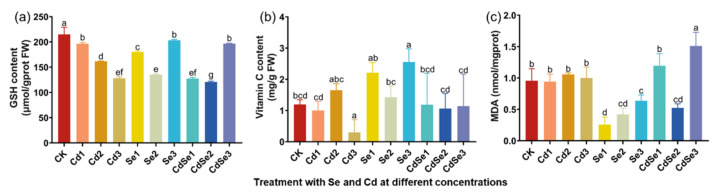
Changes in antioxidant content in mustard with different concentrations of Se and Cd. The error bar represents the standard deviation. The lowercase letter a to g on the bar reflects the 5% significance level. (**a**) GSH content of seedlings; (**b**) vitamin C content of seedlings; and (**c**) malondialdehyde content of seedlings.

With increasing Cd concentrations, the GSH content in seedlings decreased gradually (Figure 5a). However, the GSH content of seedlings was significantly increased when Se was treated alone or with Cd. When the concentration of Se and Cd was at CdSe3 levels, the GSH content of seedlings was significantly higher than that of the Cd2 treatment alone.

High-concentration Cd (Cd3) treatment significantly reduced the content of vitamin C (Vc) in seedlings, while Se treatment significantly increased the content of Vc in seedlings (Figure 5b). When Se and Cd were treated together, the Vc content of seedlings remained at the same level as that of the control. It indicated that Se treatment alleviated the decrease in Vc content in seedlings caused by Cd stress.

Cd treatment had little effect on the MDA content of seedlings, while Se treatment significantly reduced the MDA level of seedlings, among which the Se1 treatment produced the lowest MDA content (Figure 5c). Compared with Cd treatment alone, Se and Cd treatment (CdSe2) decreased the level of the MDA content, while the CdSe3 treatment significantly increased the content of MDA in seedlings. It showed that a low concentration of Se reduced the damage of Cd to cells, while a high concentration of Se and Cd damaged cells, and leads to an increase in the MDA content in cells.

### 2.5. The Content of Cd and Se in the Shoots and Roots of Seedlings

**Figure 6:**
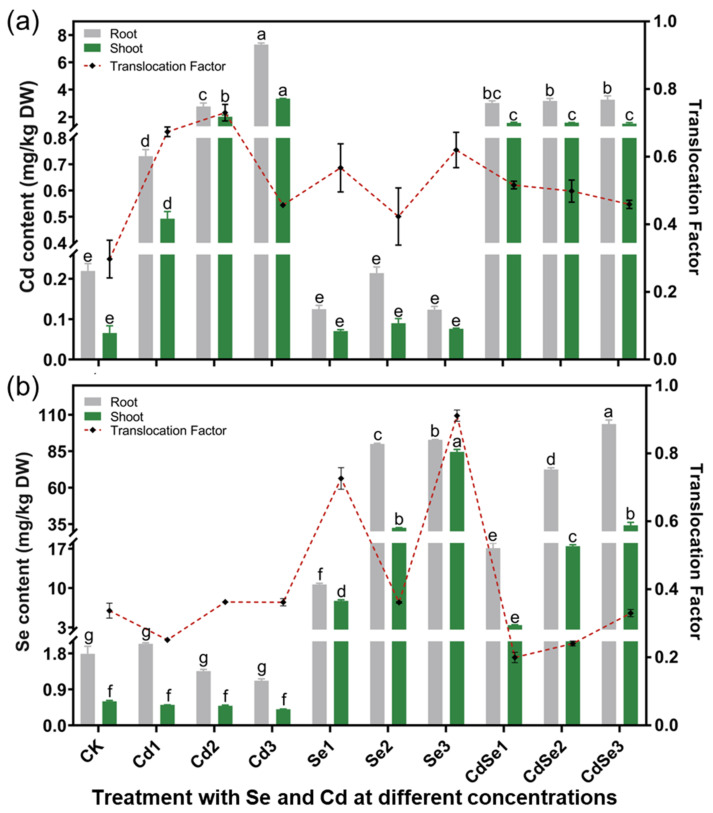
Content distribution and transfer efficiency of Se and Cd in the shoots and roots of *B. juncea* after treatment with different concentrations of CdCl_2_ and Na_2_SeO_3_. The error bar represents the standard deviation. The lowercase letter a to g on the bar reflects the 5% significance levels. (**a**) The content and transfer rate of Cd in the shoots and roots; (**b**) the content and transfer rate of Se in the shoots and roots.

When Cd was treated alone, the Cd content in the roots and the aboveground seedlings increased with increasing Cd treatment concentrations (Figure 6). The detectable Se concentration in the soil gradually increases with the increase in treatment concentration. The forms of cadmium in the soil mainly exist in water-soluble and ion-exchange forms. The proportions of Cd in Cd2, CdSe1, CdSe2, and CdSe3 in the different treatments are 67.21%, 68.95%, 66.80%, and 65.25%, respectively (Table S2). When the Cd treatment concentration was at Cd2 levels, the Cd content in the roots of seedlings reached 2.759 mg/kg, in the shooting part the Cd concentration reached 2.012 mg/kg, and the ratio between the shoot and root was 72.92%. When the Cd treatment concentration was at Cd3 levels, the Cd content in the root reached 7.309 mg/kg, in the shooting part it reached 3.339 mg/kg, and the ratio of the shoot to root was 45.69%. When the concentrations of Se and Cd were at CdSe1, CdSe2, and CdSe3 levels, the Cd content in the roots of seedlings was 3.019, 3.17, and 3.269 mg/kg, respectively. The proportion of Cd in the shoot and root was 51.59%, 49.74%, and 45.86%, respectively, which was significantly lower than 72.92% in the Cd2 alone treatment (Figure 6a). The results showed that with increasing Se concentration, Cd was mostly confined to the roots of seedlings.

With increasing Se concentration, the Se content in the shooting part of seedlings increased gradually. At Se concentrations at the Se3 level, the content of Se in the shoots and roots was 84.581 mg/kg and 92.802 mg/kg, respectively, and the proportion of Se in the shoots and roots is 92.801%. When the concentration of Se and Cd were at CdSe3 levels, the Se content in the shoots and roots was 34.235 mg/kg and 103.671 mg/kg, respectively, and the proportion of Se in the shoots and roots was 33.02% (Figure 6b). The results showed that in the presence of Cd, most Se remained in the roots of seedlings.

**Figure 7:**
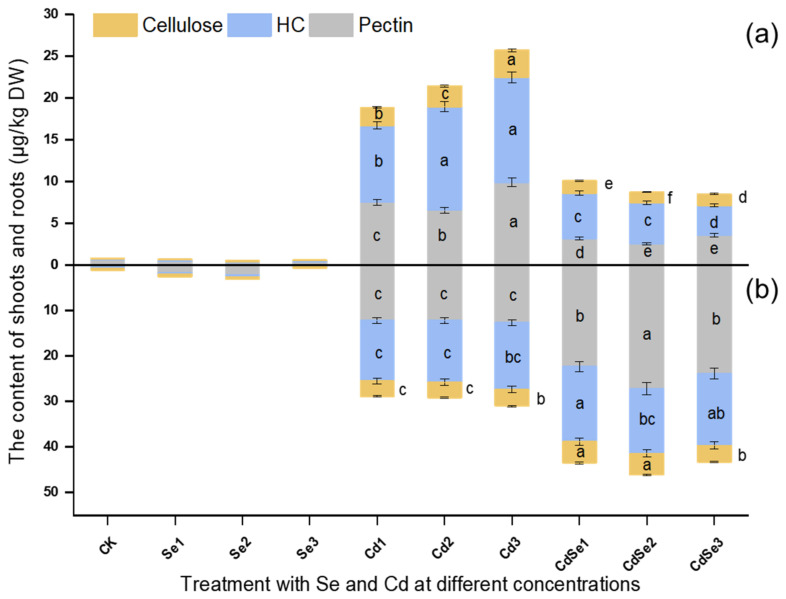
The distribution of Cd in different components of the CW of *B. juncea* in the shoots and roots after treatment with different concentrations of CdCl_2_ and Na_2_SeO_3_. Cellulose—the Cd content in the cellulose of the CW; HC—the Cd content in the hemicellulose of the CW; pectin—the Cd content in the CW of pectin. The error bar represents the standard deviation. The lowercase letter a to f on the bar reflects the 5% significance level. (**a**) Distribution of Cd in the CW of the shooting part; and (**b**) distribution of Cd in the CW of the root part.

There was a significant difference in the distribution of Cd in the CW components of the shoots and roots (Figure 7). When Cd was treated alone, Cd was mainly concentrated in the hemicellulose component of the CW. The highest concentration of Cd in the root was 14.762 mg/kg, 1.17 times that of the shoot CW part (Figure 7a). Secondly, in the pectin of the root CW, the highest Cd content reached 12.657 mg/kg, 1.28 times that of the shooting part, while the relative content of cellulose was low. When Se was added, Cd was mainly distributed in root CW, and the content of Cd in the CW pectin was significantly higher than that in the shooting part, reaching 27.18 mg/kg, 10.69 times as much as that in the shoot. The second was the hemicellulose of the root (Figure 7b). The highest content of Cd in the root was 23.913 mg/kg, which was 6.72 times that in the shoot. It indicated that pectin and hemicellulose components in the CW of root cells retained most of the Cd when seedlings were treated with Cd and Se.

### 2.6. Effects of Cd and Se on the Se Forms in Seedling

**Figure 8:**
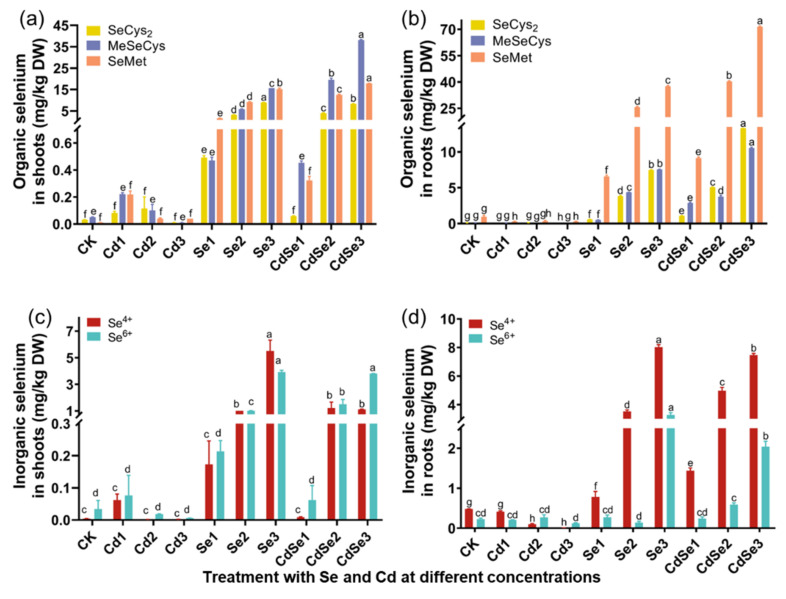
Changes in Se species contents in seedlings after treatment with different concentrations of CdCl_2_ and Na_2_SeO_3_. The error bar represents the standard deviation. The lowercase letter a to h on the bar reflects the 5% significance level. (**a**) Changes in the content of three organic Se in the shooting part; (**b**) changes in the contents of three organic Se in the root part; (**c**) changes in the contents of two inorganic Se in the shooting part; and (**d**) changes in two inorganic Se contents in the root.

When Se was treated alone or Cd and Se were treated together, the Se in the shoot and root of seedlings existed in the form of organic Se (Figure 8).

When Se was treated separately, the Se in the shoot existed in the form of SeMet, MeSeCys, and SeCys2, and the organic Se in the root mainly existed in the form of SeMet (Figure 8a,b). Inorganic Se in the aboveground part existed in the form of Se (IV) and Se (VI), and inorganic Se in the root mainly existed in the form of Se (IV) (Figure 8c,d).

When Se and Cd were treated together, the organic Se in the shoot mainly existed in the form of MeSeCys, followed by SeMet and SeCys2. When the Se concentration was at CdSe3 levels, the MeSeCys content in the shoot reached 38.119 μg/kg. The organic Se in roots mainly existed in the form of SeMet, and the Se content at CdSe3 treatment levels reached 71.453 μg/kg, followed by SeCys2, reaching 13.306 μg/kg. When Cd and Se were treated together, inorganic Se in the shoot mainly existed in the form of Se (VI), and in the root, it mainly existed in the form of Se (IV). The results showed that the content of SeMet in roots and MeSeCys in shoots increased significantly when seedlings were treated with Cd and Se.

### 2.7. Effects of Cd and Se on SA Content in Seedlings

**Figure 9:**
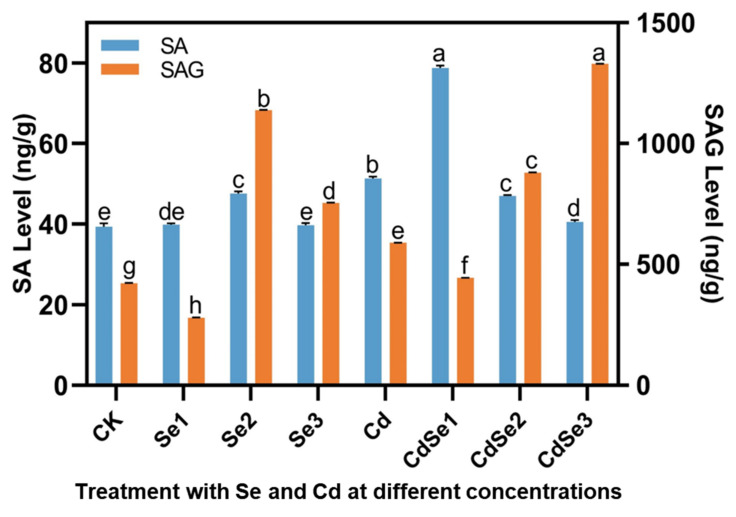
Changes of SA content in mustard with different concentrations of CdCl_2_ and Na_2_SeO_3_. The error bar represents the standard deviation. The lowercase letter a to h on the bar reflects the 5% significant level. SA, salicylic acid; SAG, Salicylate glucoside.

Salicylic acid (SA) is considered a signal molecule that mediates plant responses to abiotic stresses, such as heavy metal stress [23]. When Se was treated alone, the content of SA in seedlings increased slightly, while when the concentration was Se2, the content of Salicylate glucoside (SAG) increased significantly, reaching 1138.38 ng/g. When Cd was treated alone, SA and SAG also increased significantly, reaching 51.34 and 589.09 ng/g, respectively (Figure 9).

When the concentration of Se and Cd was CdSe1, the content of SA in seedlings reached 78.82 ng/g. When the treatment concentration was CdSe3, the SAG content reached 1330.25 ng/g (Figure 9). The results showed that low-concentration Se treatment significantly increased the content of SA in seedlings, while high-concentration Se treatment significantly increased the content of SAG. The increase in SA content was beneficial to alleviate the increase in active oxygen in seedlings under Cd stress.

### 2.8. Analysis of Transcriptome Data of Cd and Se-Treated Seedlings

In order to further analyze the effects of Se and Cd on the growth and development of *B. juncea* seedlings, The transcriptome of the treated seedlings was sequenced and analyzed. Compared with the control, there were 2674 differentially expressed genes (DEGs) in Cd-treated samples, 1637 genes were upregulated and 1037 genes were downregulated. There were 3998 DEGs in the Se1 treatment group, of which 2411 were upregulated and 1587 were downregulated, 3029 DEGs were upregulated and 889 were downregulated. There were 3029 DEGs in the Se2 treatment group, 2140 genes were upregulated and 889 genes were downregulated. There were 2543 DEGs in the Se3 treatment group, of which 2023 were upregulated and 520 were downregulated (Table S1 and Figure S2).

Compared with the control group, the CdSe2 treatment group had the most DEGs (7264), of which 5055 were upregulated and 2209 were downregulated (Figure S3a). The most DEGs among different treatments were CdSe2 and Cd1 groups, with 7698 DEGs, of which 4465 were upregulated and 3233 were downregulated (Figure S3b). The second was CdSe2 and Se2 groups, with a total of 5660 DEGs, 3419 genes upregulated and 2241 genes downregulated (Figure S3c). Compared with the control, there were 24,718 DEGs among CdSe2, Cd1, and Se2 (Figure S3d).

### 2.9. GO and KEGG Analysis of DEGs

The GO enrichment analysis of DEGs shows that, compared with the control, the DEGs treated with Cd alone were mainly enriched in the structural construction of the ribosome, CW, response to stress, oxidoreductase activity, and other functions (Figure S4a). Compared with CK, the DEGs of CdSe2 were mainly enriched in the oxidoreductase activity, response to stress, carbohydrate metabolic process, DNA binding, and other functions (Figure S4b). Compared with the Cd1 treatment, the DEGs of CdSe2 were mainly enriched in functions, such as hydrolase activity, CW organization, oxidoreductase activity, and stress response (Figure S4c). Compared with the Se2 treatment, the DEGs of CdSe2 were mainly enriched in the oxidoreductase activity, response stress, structural molecule activity, carbohydrate metadata process, CW organization, and other functions (Figure S4d).

The enrichment analysis of the KEGG pathway of DEGs showed that the DEGs treated with Cd alone were mainly enriched in the ribosome, phenoloid biosynthesis, plant path interaction, thyroid hormone signaling, and other pathways (Figure S5a). Compared with CK, the DEGs of CdSe2 were mainly enriched in the ribosome, plant hormone signal transduction, phenylpropanoid biosynthesis, starch, and sucrose metabolism, and other pathways (Figure S5b). Compared with Cd2, the DEGs of CdSe2 were mainly enriched in the ribosome, plant hormone signal transduction, phenylpropanoid biosynthesis, photosynthesis, and other pathways (Figure S5c). Compared with Se2, the DEGs of CdSe2 were mainly enriched in the ribosome, plant hormone signal transmission, phenylpropanoid biosynthesis, peroxisome, cell cycle, and other pathways (Figure S5d).

### 2.10. WGCNA Analysis of DEGs

**Figure 10:**
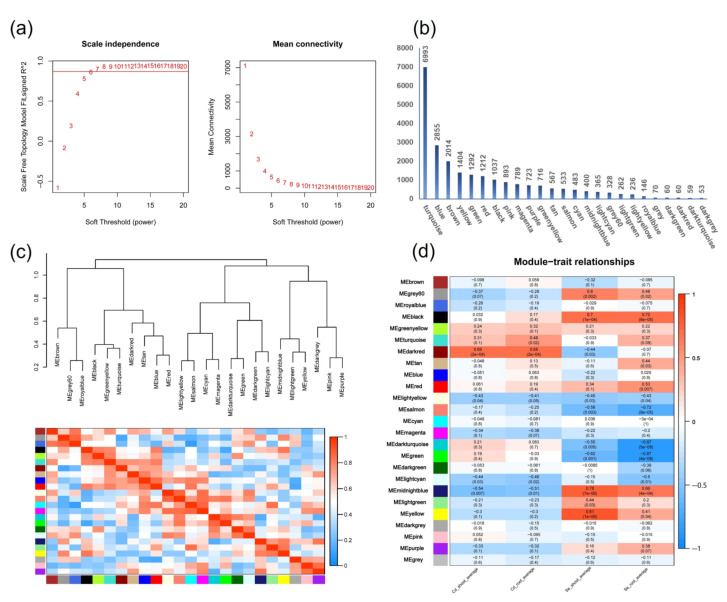
WGCNA analysis of the relationships between the aboveground and underground distribution of Cd and Se and the characteristics of transcriptome data of different treatment groups. (**a**) The scale-free network fitting index (R^2^) under different soft thresholds; the red line represents R^2^ = 0.75. The average connectivity under different soft thresholds. (**b**) The distribution and quantity of DEGs in different modules. (**c**) The WGCNA is constructed using a dynamic tree-cutting method, and different modules are marked with different colors. (**d**) The correlation between modules and traits.

**Figure 11:**
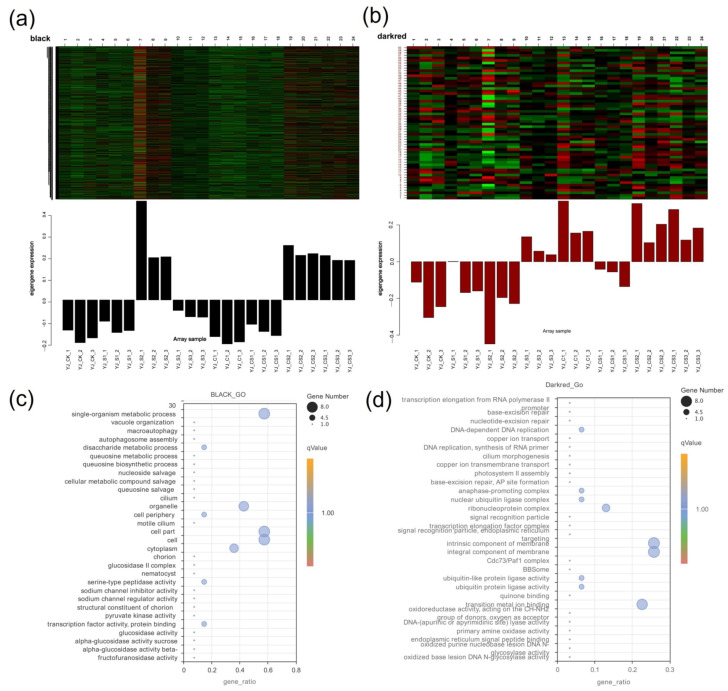
The expression patterns of genes related to Cd and Se distribution in *B. juncea* seedlings in Black and Darkred modules. In the heat map, red represents upregulated genes, black represents neutral genes, and green represents downregulated genes. Bar plots show eigengene values (i.e., the first principal component) calculated from the singular value composition. (**a**) The expression pattern of genes related to the distribution of Cd and Se in the Black module; (**b**) the expression pattern of genes related to the distribution of Cd and Se in the Darkred module; (**c**) the GO enrichment analysis of DEGs in the Black module; and (**d**) the GO enrichment analysis of DEGs in the Darkred module.

In order to further analyze the DEGs related to Se relieving Cd stress, we conducted a WGCNA association analysis on DEGs and Se and Cd content in different treatment groups (Figure 10a). WGCNA analysis finally obtained 25 modules with similar gene expression patterns, each module containing 53–6993 genes (Figure 10b,c). The correlation analysis between each module and Cd transport characteristic gene traits showed that the MEdarkred module was significantly positively correlated with Cd transport in roots and shoot parts, with correlation coefficients of (r = 0.68, *p* < 0.01) and (r = 0.69, *p* < 0.01), respectively (Figure 10d). The MEblack and MEmidlightblue modules were significantly positively correlated with Se transport in seedlings. Among them, the transport correlation coefficients of the MEblack module in the root and shoot parts were (r = 0.72, *p* < 0.01) and (r = 0.70, *p* < 0.01), respectively (Figure 10d), and the correlation coefficients between the MEmidlightblue module and the transport of Se in the root and shoot parts were (r = 0.66, *p* < 0.01) and (r = 0.78, *p* < 0.01), respectively (Figure 10d). The results showed that co-expressed genes were significantly related to the distribution of Cd and Se in the shoots and roots, and played an important role in the transport of Cd and Se. Because the MEmidnightblue module genes only participate in the differential expression of Se-treated samples alone, the MEblack module (Figure 11a) and MEdarkred module (Figure 11b) were selected for further analysis.

### 2.11. Correlation Network Analysis of Hot Genes

Figure 11 shows the results from the function enrichment analysis performed on DEGs of the MEblack module and MEdarkred module (Table S3). The results showed that the DEGs in the MEblack module were mainly enriched in cell parts, the cells, cytoplasm, and single organism metadata process functions (Figure 11c). The MEdarkred module function genes were mainly enriched in the functions of the intrinsic component of the membrane, an integral component of the membrane, transition metal ion binding, and ribonucleoprotein complex (Figure 11d). The study continued to analyze the correlation of the first 60 hot genes in the two modules. The results of morphological correlation analysis of Cd and Se in the shoot and root showed that in the MEdarkred module, the genes related to the CW and membrane included Cluster-6475.17354 (*RPS21*) and Cluster-6475.18032 (*NRT1*) in the transmembrane transporter activity, Cluster-6475.16421 (*At2g40270*) in the protein physiology, Cluster-6475.23436 (*CNGC9*) in the ion transport protein/IQ calmodulin-binding motif, Cluster-6475.17527 (*FLCY*) and Cluster-6475.17754 (*PLAT/LH2*) participate in the oxidation activity, and Cluster-6475.21245 (*PSB27-1*) participates in photosystem II repair product PSB27-H1.

**Figure 12:**
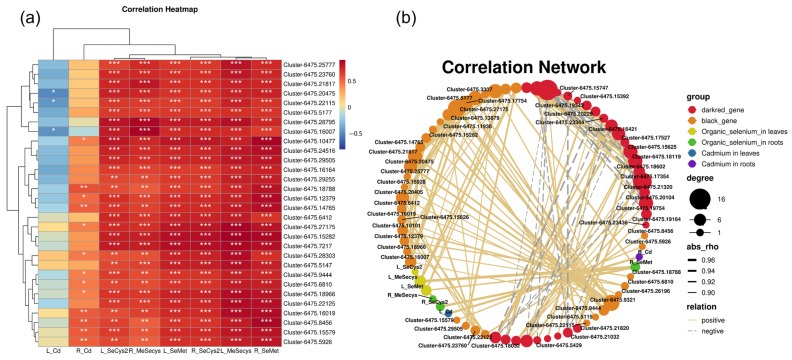
The correlation heat map and network relationships between gene expression patterns and Se forms and Cd contents in Black and Darkred modules. (**a**) Heat map of the correlation coefficient between different Se forms and Cd contents and module characteristic genes; red and blue blocks represent positive and negative correlation, respectively. The white asterisk represents a significant analysis of correlation, where * represents <0.5, ** represents <0.1, and *** represents <0.05. (**b**) Regulating network between the selected hot genes and different Se forms and Cd compounds in *B. juncea* seedlings. The positive correlation threshold was set to be greater than or equal to 0.5, the negative correlation threshold was set to be less than or equal to −0.5, and the *p*-value threshold was less than 0.5, R version 3.6.1, igraph1.2.6.

In the MEblack module, the main hot genes enriched in the DEGs related to Se alleviating heavy metal stress (Table S4). Among them, the Cluster-6475.10477 (*DHAPS-1*) gene was related to the aromatic amino acid family biological process, the Cluster-6475.21817 gene (*PME3*) was related to the CW modification/pectinesterase, the Cluster-6475.29505 gene (*SAMS2*) was related to S-adenosylmethionine biological process/methionine adenosyl-transfer activity; Cluster-6475.18788 (*4CL1*) was related to lignin synthesis and transmembrane transporter activity; and Cluster-6475.20475 gene (*DCT1*) was related to the differential transport. Cluster-6475.12379 (*NAD-ME2*) and Cluster-6475.23760 (*UCCR1-1*) genes were related to the oxidation–reduction process. In addition, in this module, Cluster-6475.22024 (*ABCC* family10), Cluster-6475.19419 (*ABCG39*), and Cluster-6475.7405 (*ZIP transporter* 4) genes were related to Cd transporters. Cluster-6475.19916 (*CAL1*) was related to a defensin-like protein and was involved in the secretion of Cd from cells to cells (Figure 12).

The above ten representative key genes were selected for qRT-PCR validation to verify the reliability of transcriptome data. The primer sequence is shown in Supplementary Table S5, and the validation results are shown in Figure S6. The results of qRT-PCR showed that the change in gene expression was basically consistent with that of RNA-Seq, indicating that the transcriptome data were highly reliable.

## 3. Discussion

### 3.1. Se Alleviates the Damage from Cd on Seedling Growth and Development

Cd has a negative effect on plant growth, and its toxicity can be observed at the morphological and physiological levels [3,20]. In winter wheat, Cd stress significantly reduced stem and root dry matter weights, root length and volume, root effective surface area, total root surface area, net photosynthetic rate, stomatal conductance, transpiration rate, and intercellular carbon dioxide amounts. Cd stress suppressed root growth and leaf photosynthesis in winter wheat, and a sufficiently elevated Se level reduced the Cd toxicity by increasing root growth [24]. Under Cd stress, with the increase in Se supply, the root length, root volume, effective root surface area, and total root surface area of winter wheat increased significantly. It may be that low-level Se increases the length of the primary root and the number of lateral roots by increasing the concentration of auxin in roots [25]. In addition, the positive effect of Se on carbohydrate metabolism will ultimately help promote plant growth [26]. In this study, the DW and FW of seedlings decreased and the root length significantly shortened after Cd treatment. However, after the addition of Se, the root length and other biomass were alleviated, and root hairs also significantly increased (Figure S1), which effectively alleviated the transport of Cd to the aboveground part, thus alleviating the damage from Cd on the plant.

Se treatment, with the appropriate concentration, can reduce the damage of Cd to plant cells and repair the function of proteins. Wheat growing in Cd-contaminated soil was treated with 20–40 mg/L Na_2_SeO_3_. With the increase in treatment concentration, the Cd content in the aboveground part of the plant was significantly reduced, and the photosynthesis of the plant was improved [27]. Se also has a similar mitigation effect on the Cd pollution of *Zea mays*. For example, Zhang et al. [28] pointed out that Se fertilizer may repair the Cd-contaminated soil entering the edible part of maize by reducing the absorption and transportation of Cd. Cd first suppresses the photosynthetic system, especially the light-harvesting complex II and both photosystems (PSI and PSII). Such damage might reduce chlorophyll and carotenoid levels [29]. Se treatment can alleviate the chloroplast dysfunction caused by Cd stress, reconstruct the ultrastructure of chloroplasts, reconstruct the thylakoid and matrix structure, and increase the content of plant chlorophyll [15,30]. In this study, the content of chlorophyll B in seedlings decreased significantly when Cd was used alone, but increased after Se was added.

Altered root morphology and suppressed root growth are broadly considered early responses of plants to environmental stressors such as heavy metals and nutrient shortages [24,31]. Some studies have shown that Cd exposure significantly reduces root cell activity, root length, and root biomass. It has been reported that Cd shows a negative effect on root morphology by inhibiting cell division and elongation, resulting in a reduction in root length, volume, average diameter, and surface area of plants [18]. This inhibition of root growth has been proven to be one of the obvious symptoms of Cd toxicity, which may be caused by the low mitotic activity of meristem cells under Cd stress [32]. In addition, the reduction in root length, surface area, specific root length, and root tip number are equally related to Cd stress, indicating that the plant’s ability to acquire resources (e.g., water and nutrients) is reduced in the case of Cd stress. Therefore, the morphological features of the root are considered key indexes for assessing Cd toxicity [33]. The research of Qi et al. [3] showed that foliar spray containing Na_2_SeO_3_ or nano Se can significantly reduce root tip damage (especially the apical meristem) induced by Cd, restore root cell vitality of the meristem, and reduce the Cd inhibition of root elongation.

### 3.2. Se Improves the Adsorption Capacity of Root CW to Cd

The CW represents the first barrier in preventing toxic molecules from entering the cell, and the binding site of major heavy metals, e.g., Cd [7]. The CW of vascular plants forms a physical barrier to toxic elements by interacting with negatively charged functional groups, and pectin, cellulose, and hemicellulose play an important role in such interactions [34]. Recently, Wan et al. [35] demonstrated that hemicellulose was usually at the site of heavy metal binding, and the protocell wall may be the main site of polysaccharide binding in dicotyledonous plants. A further constituent was pectin which comprises 70% of negative charges and enables Cd binding. Through long-term exposure to Cd, the CW changes, leading to CW metabolism and lignification remodeling. Therefore, high polysaccharide amounts in root CWs can accommodate more Cd [36]. The adsorption capacity of pectin, hemicellulose, and cellulose to Cd varies with plants. For example, in *Arabidopsis*, the amount of pectin, hemicellulose, and cellulose that can adsorb Cd accounts for 23%, 56%, and 21%, respectively [37], while in rice, the amount of pectin and hemicellulose in the CW can adsorb 71–85% of Cd in roots [38]. Yu et al. reported that Cd-tolerant rice varieties isolated more Cd in CWs and vacuoles. Therefore, compared with sensitive rice varieties, the Cd binding capacity of the CW and pectin in Cd-tolerant rice was stronger, which limited its transfer from the roots to shoot parts [38].

The study of Zhao et al. showed that Se increased the content of CW polysaccharide, changed the subcellular distribution and chemical form of Cd, and was conducive to the fixation of Cd in the root [18]. In addition, exogenous Se increased the content of pectin and hemicellulose in the CW of *Brassica napus* root. These studies showed that Se’s effect on pectin and hemicellulose quantities enhanced the interaction of Cd and the CW, thus altering the Cd distribution in cells. Moreover, Se increased the CW thickness and lignin levels by regulating genes associated with Cd and lignin synthesis to strengthen the CW and decrease Cd flow into plant cells [20]. In this study, when Cd was administered alone, it was mostly found in the hemicellulose of root CWs. After adding Se, Cd was mostly detected in the pectin and hemicellulose of root cells. These findings showed that Se treatment effectively enhanced Cd adsorption and isolation by root pectin.

### 3.3. Se Alleviates ROS Produced by Cd Stress

Cd pollution in the soil causes serious toxicity to plant growth, such as excessive production of ROS, while a certain amount of ROS will also be produced during normal metabolic growth. Plants eliminate ROS through the antioxidant system, which includes antioxidant enzymes (SOD, POD, and CAT) and antioxidant compounds (ASA and GSH) [39]. Under physiological conditions, an equilibrium exists between antioxidants and ROS. It was suggested that Se reduces Cd-induced oxidative damage thanks to its antioxidant properties [40]. SOD converts O_2_^•−^ into hydrogen peroxide, which is converted by POD and CAT into O_2_ and H_2_O [41]. Therefore, increased SOD and POD activities or the upregulation of respective proteins might be key to maintaining the ROS balance of the plant under Cd stress, thereby improving Cd tolerance. In addition, the GSH–ASA cycle regulates the oxidoreduction steady state by eliminating hydrogen peroxide [42]. Here, Cd markedly increased the activities of the main antioxidant enzymes in seedlings to cope with a large amount of ROS produced by Cd stress.

Many studies have shown that Se supplementation regulates ROS metabolism and MDA by strengthening the protective system and remarkably clearing the active oxygen species, thus reducing oxidative stress resulting from heavy metals [7]. The hydroponic experiment involving rice seedlings revealed that Na_2_SeO_3_ and Na_2_SeO_4_ can reduce the damage of hydrogen peroxide and MDA associated with Cd stress by improving the plant’s antioxidant system [43]. Exogenous selenite significantly reduced oxidative stress induced by Cd in rice and enhanced SOD, POD, GR, and APX activities in plant tissues [44]. In a report on Cd stress in wheat, NADPH oxidase expression in Cd-tolerant varieties was elevated compared to that of common varieties, while ROS and MDA contents were lower, suggesting that the antioxidant system of Cd-tolerant varieties was more efficient in removing ROS produced by Cd stress, thereby reducing oxidative damage [42]. In this research, compared with Cd treatment alone, the activities of the main antioxidant enzymes and the amounts of the antioxidant GSH and Vc decreased significantly after Se addition, indicating that Se effectively alleviates Cd-induced oxidative stress.

Cell membrane peroxidation and damage associated with ROS yields MDA, which reflects the extent of cell membrane oxidation [42]. MDA production increases cell membrane damage in plants. Se can reduce the concentration of MDA in Chinese cabbage, thus alleviating cell membrane damage under Cd stress. Proper concentrations of Se can increase the contents of unsaturated fatty acids as well as the fluidity of cell membranes and the reactivation of membrane enzymes to restore the transport of critical metabolites, thus decreasing Cd stress in plants [30]. In this study, the CdSe2 treatment effectively reduced the plant content of MDA, while the CdSe3 treatment increased the content of MDA. High Se and Cd amounts might cause greater damage to the plant.

### 3.4. Se Amino Acids Reduced the Cd Content in the Shoots of Seedlings

GSH is also an important antioxidant, which can reduce ROS in cells to reduce plant damage. It is believed that GSH directly interacts with Se to produce SeMet and selenocysteine (SeCys) and to synthesize Se-rich proteins [45]. Wan et al. [43] suggested that Se supplementation increased GSH to offset Cd toxicity. SeCys represents a critical part of the glutathione peroxidase (GSH-Px)’s activity center. GSH-Px catalyzes GSH production. GSH interacts with ascorbic acid (ASA) to break down hydrogen peroxide via the Halliwell–Asada pathway, thus preventing ROS-induced structural and functional cell damage [46].

Filek et al. believed that Se alleviated the toxicity of Cd by competing with the sulfhydryl group of cysteine [30]. In several plants, Se produces more glutathione (GSH) under Cd stress. Adding Se to Chinese cabbage can significantly immobilize Cd, indicating that SeCys/SeCys2 plays an important role in reducing Cd during plant growth [19]. In the shooting part of *B. juncea*, after Se treatment, the Cd content of the aboveground part was much lower than that of the Cd treatment alone. It is speculated that this may be related to the increase in the content of SeCys, resulting in a large amount of GSH and Cd immobilization.

Yu et al. speculated that Cd and Se were likely to be transported in the root of *Brassica chinensis* in the form of CdSeO_3_^0^ and CdSeO_4_^0^ [17]. A Se–Cd complex has no bioavailability to plants; however, plants show a significant reduction in Cd absorption [28]. The research and analysis showed that SeCysCysSe could exist in soil with a low Se:Cd (<0.7) ratio in the leaves at the same time, but when the Se:Cd (>0.7) ratio was high, Se bound Cd in the form of SeMet, indicating that Se–Cd compounds existed in the leaves and had direct interaction [7]. Vascular plants use Se through sulfur assimilation. Relatively rich Se can be converted into selenides and enter amino acids and proteins. In cells, Cd is often connected with sulfur ligands, especially mercaptan groups, such as plant chelating proteins (PCs) and glutathione (GSH), and more importantly, SeH, such as SeCys, GSH-PCs, and other forms [47,48]. In this study, a large amount of Se in the root was converted into SeMet, and the increase in SeMet content effectively reduced the transport of Cd to the shooting parts.

### 3.5. Salicylic Acid and Cd Stress

In plants, SA is a ubiquitously detected phenolic hormone, which alleviates Cd toxicity by controlling plant growth, and decreases Cd absorption and distribution, thus preserving membrane integrity and stability, clearing ROS, strengthening the antioxidant defense system, and enhancing photosynthetic capacity [49]. In pepper, 1 mg/L and 5 mg/L nanoSe treatments significantly increased the content of SA in leaves [50]. The change in plant SA levels induces the recovery of root damage. For instance, the SA (10 µM) treatment of chickpea plants after exposure to low, moderate, and strong Cd stress results in the increased restoration, restoration, and partial restoration of root lengths, respectively [51]. It can be inferred that increased SA levels in *B. juncea* are tightly associated with the recovery of roots and the growth of root hairs.

Under Cd stress, SA can increase the content of pectin in the CW and reduce the degree of methyl esterification of pectin [23]. SA treatment increased the accumulation of Cd in peanut CWs and reduced the distribution of Cd in organelles [52]. In another study, the growth of rice roots was markedly suppressed by Cd, and seedling leaves showed severe dehydration, while SA application alleviated the above inhibitory effects [53]. Yotsova’s study suggested that the exogenous application of 10 μmol/L SA via the rooting medium prevents Cd toxicity in rice plants [54]. In addition, a study revealed that SA application decreased Cd transport from root to stem and increased the activities of antioxidant enzymes of plant root and stem [55]. In plants, the hydroxyl group of SA interacts with glucose to form SA glucoside. SAG is transported from the cytoplasm to vacuoles and is stored in an inactive form. When the plant is under stress, it can release free SA [56]. Here, SA and SAG contents were significantly higher in seedlings treated with Cd and Se than those treated with Cd alone, and both had a dynamic regulation. The increased SA amount may be associated with increased pectin content in root CWs, which is critical for the adsorption of some Cd on root CWs.

### 3.6. Se Regulates the Expression of Genes Related to Cd Stress

Studies have reported that Se plays a role in regulating key Cd transporter genes in rice. Se can downregulate genes associated with Cd transport. As a mechanism to alleviate the toxicity of Cd, Se promotes the biosynthesis of lignin, thereby reducing the bioavailability of Cd [20,57]. In the Black and Dark modules of this study, Cluster-6475.18788 (*4CL1*) participated in lignin synthesis and had a significant negative correlation with Cd content in the aboveground part. It is speculated that this may be related to the promotion of lignin synthesis by the *4CL* gene and reduced Cd transport to the aboveground part.

Studies have shown that ABCC, ZIP, and Nramp family transporters play major roles in Cd transport and tolerance in *Hibiscus cannabinus* [41]. In rice, the OsNramp5 protein is located at the distal end of the outer epithelial and endothelial layers of root cells, with polarity. OsNramp5 mediates Mn uptake and transport and is also the main Cd transporter in rice [58]. Cui et al. showed that Se supplementation downregulates *OsNramp5* and *OsLCT1* and upregulates *OsHMA3* to promote the isolation of Cd in cell vacuoles [20]. Studies suggested that iron transporters (OsIRT1, OsIRT2, and OsNRAMP1) also perform Cd uptake [9]. In addition, in wheat, ABCC subfamily members also participate in the detoxification of Cd through vacuole isolation [42]. In soybean, NRT family proteins may participate in SeMet transport to the aboveground part [59]. *NRT1.1B* overexpression in rice markedly increased SeMet transport from root to shoot, causing an increase in Se concentration in shoot and grain [60]. In this study, the bivalent cation transport protein DCT1 participated in the induced expression of Se under Cd stress, which may be related to Cd isolation and transport in root cell vacuoles. The *NRT1* gene was involved in the transport of Se to the aboveground part.

Other critical metal transporters in plants are heavy metal ATPases (HMAs). In rice, OsHMA3 was a transporter located on the vacuolar plastid, while OsHMA2 was found on the cytoplasmic membrane. They participate in Cd and Zn transport [12]. *OsHMA2* gene deletion reduced Cd content in the shoot of the plant [61]. OsHMA3 mediates Cd isolation in root vacuoles. *OsHMA3* gene deletion enhanced the absorption and accumulation of Cd into stems and grains [10]. The overexpression of *OsHMA3* decreased Cd transport from the root to the aboveground part [62]. In rice, OsLCT1 (low-affinity Cd transporter 1) constitutes a cell membrane transporter. Knocking out *OsLCT1* in plants decreased Cd amounts in phloem and grain. Luo et al. discovered a new defensin-like protein (OsCAL1) in rice, which can control the content of Cd in leaves, promote the secretion of Cd into the extracellular space through cytoplasmic chelation, and reduce the content of Cd in the cytoplasm [63]. CAL1 is a Cd^2+^-binding protein, which controls Cd^2+^ amounts in rice [64]. In *B. juncea* treated with Se and Cd, the transcriptional downregulation of Cluster-6475.7405 (*ZIP transporter* 4) may be related to decreased Cd transport to the shooting part. The upregulation of Cluster-6475.19916 (CAL1) may be related to the cytoplasmic chelation of Cd.

## 4. Materials and Methods

### 4.1. Treatment of Plant Materials

*B. juncea* underwent hydroponic culture in a greenhouse at 25/20 °C (day/night) under a 12 h photoperiod, with 70% relative humidity and 300–380 μmol (m^2^s)^−1^ of light. *B. juncea* seeds were rinsed thoroughly with deionized water, sterilized on the surface using 1% mercuric chloride for 8 min, and treated with CaSO_4_ for 4 h before 10-day germination on vermiculite. The pH value of the vermiculite culture medium was 7.0, and no Cd and Se were detected in the vermiculite by inductively coupled plasma mass spectrometry (ICP–MS 8900, Agilent Technologies, Santa Clara, CA, USA). After 4 days of germination, seedlings underwent transplantation into 1.5 L plastic tanks with 1/5 Hoagland solution [65]. The composition of the nutrient solution was 1.0 mM Ca(NO_3_)_2_, 1.0 mM KNO_3_, 0.4 mM MgSO_4_, 0.5 mM NH_4_H_2_PO_4_, 0.03 mM EDTA-Fe, 3 μM H_3_BO_3_, 1.0 μM MnSO_4_, 0.1 μM ZnSO_4_, 0.2μM CuSO_4_, and 0.2μM Na_2_MoO_4_. The nutrition solution (pH 5.8) was buffered with 2-morpholinoethanesulfonic acid and renewed every 3 days according to Yu’s method [20]. The treatment concentration of Na_2_SeO_3_ was set to 10, 50, or 100 mg/kg; that of CdCl_2_ was 10, 50, or 100 mg/kg. The CdCl_2_+ Na_2_SeO_3_ groups were treated with 50 + 10, 50 + 50, and 50 + 100 mg/kg, and the control group was incubated in the vermiculite medium with 1/5 Hoagland solution. After 10 days of incubation in vermiculite, sample collection was carried out. After the samples were washed with distilled water, some roots and leaves were immediately collected and stored at −80 ℃ for subsequent index determination; others underwent drying to constant weight for the assessment of element contents, Se form, etc.

After collecting the plant samples, a regular quadrilateral shape on the surface of the potted soil was made, and the soil at each corner of the regular quadrilateral shape was taken. After the soil was air-dried, it was sieved to remove plant roots and stems and then subjected to a “medium crushing–shrinkage–fine crushing” process. To a soil sample of 2.500 g, a quantitative extraction solution was added, shaken, centrifuged, filtered, separated, and, finally, the Se, Cd, and form contents were determined using cold atomic fluorescence spectrometry. The measurement procedure was completed according to Zeng’s method [66].

### 4.2. Microstructural Observations of Root Tissue

Primary root tips (approximately 2 cm long) underwent a 10 min incubation with propidium iodide (PI) solution at 25 ℃ before three rinses with deionized water. After staining, roots were examined under a laser scanning confocal fluorescence microscope (Olympus, SpinSR10, Tokyo, Japan) at excitation and emission wavelengths of 485 and 530 nm, respectively. Fresh roots underwent washes with 0.5 mM CaCl_2_ (pH 4.5) and drying using filter papers. This was followed by a 30 min staining with 5 mL of magenta solution (0.025%, *w/v*; pH 5.5), three rinses with distilled water, and analysis under a light microscope (SZHILLD, Nikon, Kyoto, Japan).

### 4.3. Determination of Phytohormone in B. juncea

The phytohormone content of *B. juncea* root and shoot was determined using the Metware Biotech Co., Ltd. LC–MS/MS platform (Metware Biotech Co., Ltd., Wuhan, China, www.metware.cn, 5 September 2022). The API 6500 Q TRAP LC/MS/MS System, coupled to an ESI Turbo Ion Spray interface was run in the positive ion mode utilizing Analyst 1.6 (AB Sciex, Toronto, ON, Canada), as follows: an ion source, turbo spray; source temperature, 500 °C; ion spray voltage, 5500 V; curtain gas, 35.0 psi; collision gas, medium level. DP and CE for each MRM transition were optimized. Specific MRM transitions were monitored for various periods based on eluted phytohormones.

### 4.4. Determination of Cd and Se Contents in B. juncea

About 0.3 g of dry root specimen was utilized for measuring Cd uptake kinetics; 0.60 g of freshly collected shoots and 0.35 g of freshly obtained roots underwent digestion in a microwave oven (Midea PM2000, Guangdong, China) in 8 mL of 65% HNO_3_ at 180 °C for exposure assays. The concentrations of these elements were obtained by inductively coupled plasma optical emission spectrometry (Optima 7300 DV; PerkinElmer, Waltham, MA, USA) for uptake kinetics, and ICP–MS (8900; Agilent Technologies, Santa Clara, CA, USA) for exposure experiments. Calibration standards for 111Cd and 78Se (1000 mg L^−1^ in 2% HNO_3_) were provided by the National Institute of Metrology of China (Beijing, China). Cd and Se amounts were assessed at 228.80 and 196.02 nm, respectively. Both ICP–OES and ICP–MS utilized argon with >99.99% purity. Plasma, auxiliary, and nebulizer gas flow rates were 15, 0.2, and 0.6 L/min for ICP–OES, respectively, and 13, 0.8, and 0.8 L/min for ICP–MS, respectively. Digestion blanks and CK mustard were simultaneously detected as controls. The control recovery rates were 85–115%.

Se speciation in *B. juncea* was determined using the LC–HG–AFS method, detecting Se (IV), Se (VI), selenocysteine (SeCys2), methyl selenocysteine (MeSeCys), and SeMet. The calculation formula of Se content for each form in seedling specimens was (mg/kg): (C × V × N)/(W × 1000), where C is the mass concentration of the Se form (μg/L), V is the total volume of the solution to be tested (mL), N represents the dilution factor, and W represents the dry weight of the sample (g).

### 4.5. Determination of Antioxidant Enzyme Activity and Hydrogen Peroxide, Glutathione, Ascorbic Acid, and Malondialdehyde Contents

Freshly collected shoots underwent homogenization with phosphate-buffered saline (pH 7.3), followed by centrifugation at 1126 g for 10 min. The resulting supernatants were assessed for the amounts of H_2_O_2_, MDA, and all antioxidants. Catalase (CAT), peroxidase (POD), superoxide dismutase (SOD), glutathione reductase (GR) activities, and glutathione (GSH), ascorbic acid (ASA), and malondialdehyde (MDA) levels in shoots treated with Cd for 10 days were assessed with specific kits (Jiancheng Bioengineering Institute, Nanjing, China). Specific models are as follows: the A045-2-2 kit (Coomassie brilliant blue method) was used for the determination of total protein, the A005-1-2 kit (colorimetry) was used for the determination of glutathione peroxidase (GSH-PX), the A006-2-1 kit (microplate method) was used for the determination of reduced glutathione (GSH), the A004-1-1 kit (colorimetry) was used for glutathione-S transferase (GSH-ST), the A001-1-2 kit (hydroxylamine method) was used for total superoxide dismutase (T-SOD), and the A003-1-2 kit (TBA method) was used for the determination of malondialdehyde (MDA).

### 4.6. Transcriptome Sequencing and Analysis

Total RNA at each stage was obtained with the TRIzol Kit (Promega, Beijing, China), as directed by the manufacturer. After treatment with DNase I (Takara), RNA integrity and quality were confirmed by RNase-free agarose gel and NanoDrop 2000 (Implen, Westlake Village, CA, USA).

Index-coded specimens were clustered on a cBot Cluster Generation System using TruSeq PE Cluster Kit v3-cBot-HS (Illumina), as instructed by the manufacturer. Upon clustering, the generated libraries underwent sequencing on an Illumina Novaseq platform to produce 150 bp paired-end reads. Raw reads (FASTQ format) first underwent processing utilizing in-house perl scripts. Q20, Q30, and GC contents of clean reads were determined. All subsequent analyses utilized clean reads of high quality. Reference genome and gene model annotation files were downloaded from the genome website directly. Mapped reads for various samples were assembled with StringTie (v1.3.3b, Johns Hopkins University, Baltimore, MD, USA), as proposed previously [67].

Transcriptome assembly was next carried out with Trinity v2.4 (The Broad Institute, Cambridge, MA, USA). Gene functions were annotated utilizing the National Center for Biotechnology Information nonredundant protein sequences (Nr), Protein family (PFAM), Kyoto Encyclopedia of Genes and Genomes (KEGG), the evolutionary genealogy of genes: Nonsupervised Orthologous Groups (Egg-NOG), Swiss-Prot and Gene Ontology (GO). The expression of unigenes was given as fragments per kilobase of transcript per million fragments (FPKM).

### 4.7. The WGCNA Analysis of Cd Absorption and Transport, Se Speciation, and Related Genes

In order to determine the biosynthesis pathway of Se-containing amino acids and major regulatory genes of Cd absorption and transport in response to Na_2_SeO_3_ and CdCl_2_, weighted gene co-expression network analysis (WGCNA) was performed with Novogene (https://magic.novogene.com/customer/ accessed on 15 October 2022). The module’s eigengene was its first principal component, which reflected the expression profile of the module’s genes in a given specimen. Pearson correlations between a given eigengene and flavonoids were obtained using ggplot2 in R.

### 4.8. Real-Time Quantitative PCR (qRT-PCR) Validation

qRT-PCR utilized cDNA as a template. Amplification was performed with 2×TSINGKE Master qRT-PCR Mix (SYBR Green I) and GoldenStar™ RT6 cDNA Synthesis Mix (TSINGKE, China) on an Applied Biosystems ABI 7500 (Foster City, CA USA). Primers designed with Snap Gene (v4.3.6, Table S5) were used. Baseline and threshold cycles (Ct) were calculated by the system’s software. Data analysis used Actin expression for normalization.

### 4.9. Statistical Analysis

Data analysis utilized Excel 2021 (v2212, Microsoft Corporation, Redmond, WA, USA) and SPSS (v22.0, IBM Corporation, Armonk, NY, USA). Duncan’s test was utilized for comparisons, and *p* < 0.05 indicated statistical significance. Three biological replicates were examined in each assay. Correlation analysis was carried out using OmicStudio (https://www.omicstudio.cn/tool accessed on 3 March 2023), with positive and negative correlation thresholds of ≥0.5 and ≤−0.5, respectively (*p* < 0.05), based on R version 3.6.1, igraph1.2.6.

## 5. Conclusions

**Figure 13:**
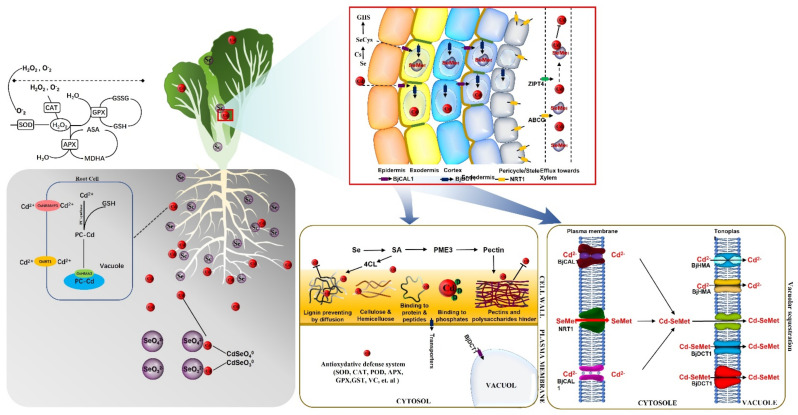
The picture shows the absorption and metabolic transport mechanism of Cd under the influence of Se in *B. juncea*. Se addition alleviated Cd inhibition of seedling biomass, root length, and chlorophyll. In this study, the mechanism of Se reducing Cd toxicity is summarized as follows: Se reduces the oxidative stress induced by Cd and reduces the content of MDA in cells; Secondly, Se enhanced the adsorption of Cd by pectin and lignin in the root cell wall by promoting SA levels; In addition, in *B. juncea*, the large amount of synthesis of SeCys and SeMet reduces the transport of Cd to the aboveground part; finally, Se treatment induced the expression of genes, such as *MPP*, *ABCC,* and *CAL1*, which was helpful to chelate and separate Cd.

Through the treatment of Cd and Se, this study summarized several possible mechanisms by which Se could reduce the toxicity of Cd to *B. juncea* (Figure 13). First, in terms of physiological indicators, Se treatment alleviated the inhibitory effects of Cd on seedling biomass, root length, and photosynthetic pigments, and reduced Cd damage to root tip cells. Secondly, Se treatment effectively enhanced Cd absorption and isolation by root pectin, and promoted the synthesis of lignin in root tip cells, reducing the Cd uptake by seedlings. Compared with Cd treatment alone, the activities of main antioxidant enzymes and the contents of key antioxidants decreased significantly after adding proper amounts of Se, indicating that Se effectively reduced Cd-induced oxidative stress and decreased MDA content in cells. In the aboveground part of seedlings, increased SeCys content produced a large amount of GSH and immobilized Cd. Elevated SeMet content in roots effectively reduced the transport of Cd to the aboveground part. Elevated SA content might be associated with increased pectin content in root CWs, which is critical for the adsorption of some Cd on root CWs. The analysis of transcriptome data showed that bivalent cation transporter DCT1 participated in the isolation of Cd in the cell vacuoles, CAL1 was related to the chelation of Cd in the cell cytoplasm, and ZIP transporter 4 reduced the transport of Cd to the shooting part.

## Data Availability

Data will be made available upon request.

## Supplementary Materials

The following supporting information can be downloaded at: https://www.mdpi.com/article/10.3390/plants12081583/s1, Figure S1: Effects of different concentrations of Se and Cd on root tip growth of *B. juncea.* (a) CK; (b) Cd1—the treatment of seedling root tips with 10 mg/L CdCl_2_; (c) Cd2—50 mg/L CdCl_2_; (d) Cd3—100 mg/L CdCl_2_; (e) Se1 treatment of seedling root tips with 10 mg/L Na_2_SeO_3_; (f) Se2—50 mg/L Na_2_SeO_3_; (g) Se3—100 mg/L Na_2_SeO_3_; (h) CdSe1—50 mg/L CdCl_2_ and 10 mg/L Na_2_SeO_3_; (i) CdSe2—50 mg/L CdCl_2_ and 50 mg/L Na_2_SeO_3_; and (j) CdSe3—50 mg/L CdCl_2_ and 100 mg/L Na_2_SeO_3_; Figure S2: The number of DEGs among samples treated with different concentrations of CdCl_2_ and Na_2_SeO_3_. The green histogram shows the total DEGs, the gray represents the number of upregulated DEGs, and the blue represents the number of downregulated DEGs; Figure S3: Volcano map and Venn map for comparison of differentially expressed genes between samples treated with different concentrations of sodium selenite and cadmium chloride: (a) volcano map of DEGs between CdSe2 and control; (b) volcano map of DEGs between CdSe2 and Cd1; (c) volcano map of DEGs between CdSe2 and Se2 treatments; and (d) Venn diagram of DEGs between CdSe2, Cd1, Se2, and control group; Figure S4: GO enrichment analysis of DEGs in samples treated with different concentrations of CdCl_2_ and Na_2_SeO_3._ (a) GO enrichment of DEGs between Cd1 and control group; (b) GO enrichment of DEGs between CdSe2 and control; (c) GO enrichment of DEGs between CdSe2 and Cd1; (d) GO enrichment of DEGs between CdSe2 and Se2 treatments; Figure S5: KEGG pathway enrichment analysis of DEGs in samples treated with different concentrations of CdCl_2_ and Na_2_SeO_3_: (a) KEGG pathway enrichment of DEGs between Cd1 and control group; (b) KEGG pathway of DEGs between CdSe2 and control; (c) KEGG pathway of DEGs between CdSe2 and Cd1; and (d) KEGG pathway of DEGs between CdSe2 and Se2 treatments; Figure S6: Comparative validation of qRT-PCR and RNA-seq of candidate genes related to Se and Cd uptake, transport, and metabolism in *Brassica juncea*. The *Actin* gene was used as a control, and the 2(^−ΔΔCt^) analysis method was used to determine the relative expression levels of genes. The error bars represent the SD of three biological replicates. The numbers above the graphics correspond to values obtained with the correlation analysis of the gene expression ratios obtained from the RNA-seq data (blue column) and the qRT-PCR data (yellow line). CK: control samples; S1 is a mustard sample treated with 10 mg/L Na_2_SeO_3_; S2 is a mustard sample treated with 50 mg/L Na_2_SeO_3_; S3 is a mustard sample treated with 100 mg/L Na_2_SeO_3_; Cd is a mustard sample treated with 50 mg/L CdCl_2_; CdSe1 is the mustard sample treated with 50 mg/L CdCl_2_+10 mg/L Na_2_SeO_3_; CdSe2 is the mustard sample treated with 50 mg/L CdCl_2_+50 mg/L Na_2_SeO_3_; CdSe3 is the mustard sample treated with 50 mg/L CdCl_2_+100 mg/L Na_2_SeO_3_; Table S1: *B. juncea* treated with different concentrations of CdCl_2_ and Na_2_SeO_3_ and the use of treated samples; Table S2: Changes in the occurrence of cadmium in the soil of mustard treated with different concentrations of Se and Cd; Table S3: Annotation analysis of the first 30 hot spot genes screened in the Darkred module; Table S4: Annotation analysis of the first 30 hot spot genes screened in the Black module; Table S5: qRT-PCR validation primer for DEGs hot genes related to Se and Cd treatment.
