# Supplementary material for: Morphological and Physiological Indicators and Transcriptome Analyses Reveal the Mechanism of Selenium Multilevel Mitigation of Cadmium Damage in Brassica juncea
Source: Plants (Basel). 2023 Apr 7;12(8):1583. doi: 10.3390/plants12081583 (PMC10141491; doi:10.3390/plants12081583)
Supplement: Supplementary file 1 [file plants-12-01583-s001.zip › plants-2299763-supplementary.pdf]

Table S1. *B. juncea* treated with different concentrations of $CdCl_2$ and Na_2SeO_3 and the use of treated samples.

Sample name	Treatment (mg/L)	Name in the article	Index determination	Sowing date	collection date
CK	0	Root of CK	Transcriptome, Phytohormone, Se content, Cd content, Se forms	2022.4.20	2022.5.12
		Shoot of CK	Transcriptome, Physiological and biochemical indexes, Phytohormone, Se content, Cd content, Se forms	2022.4.20	2022.5.12
Cd1	10 mg/L $CdCl_2$	Root of Cd1	Transcriptome, Phytohormone, Se content, Cd content, Se forms	2022.4.20	2022.5.12
		Shoot of Cd1	Transcriptome, Physiological and biochemical indexes, Se content, Cd content, Se forms	2022.4.20	2022.5.12
Cd2	50 mg/L $CdCl_2$	Root of Cd2	Transcriptome, Phytohormone, Se content, Cd content, Se forms	2022.4.20	2022.5.12
		Shoot of Cd2	Transcriptome, Physiological and biochemical indexes, Phytohormone, Se content, Cd content, Se forms	2022.4.20	2022.5.12
Cd3	100 mg/L $CdCl_2$	Root of Cd3	Transcriptome, Phytohormone, Se content, Cd content, Se forms	2022.4.20	2022.5.12
		Shoot of Cd3	Transcriptome, Physiological and biochemical indexes, Se content, Cd content, Se forms	2022.4.20	2022.5.12
Se1	10 mg/L Na_2SeO_3	Root of Se1	Transcriptome, Phytohormone, Se content, Cd content, Se forms	2022.4.20	2022.5.12
		Shoot of Se1	Transcriptome, Physiological and biochemical indexes, Phytohormone, Se content, Cd content, Se forms	2022.4.20	2022.5.12
Se2	50 mg/L Na_2SeO_3	Root of Se2	Transcriptome, Phytohormone, Se content, Cd content, Se forms	2022.4.20	2022.5.12
		Shoot of Se2	Transcriptome, Physiological and biochemical indexes, Phytohormone, Se content, Cd content, Se forms	2022.4.20	2022.5.12
Se3	100 mg/L Na_2SeO_3	Root of Se3	Transcriptome, Phytohormone, Se content, Cd content, Se forms	2022.4.20	2022.5.12
		Shoot of Se3	Transcriptome, Physiological and biochemical indexes, Phytohormone, Se content, Cd content, Se forms	2022.4.20	2022.5.12
CdSe1	50 mg/L $CdCl_2$+10 mg/L Na_2SeO_3	Root of CdSe1	Transcriptome, Phytohormone, Se content, Cd content, Se forms	2022.4.20	2022.5.12
		Shoot of CdSe1	Transcriptome, Physiological and biochemical indexes, Phytohormone, Se content, Cd content, Se forms	2022.4.20	2022.5.12
CdSe2	50mg/L $CdCl_2$+50 mg/L Na_2SeO_3	Root of CdSe2	Transcriptome, Phytohormone, Se content, Cd content, Se forms	2022.4.20	2022.5.12
		Shoot of CdSe2	Transcriptome, Physiological and biochemical indexes, Phytohormone, Se content, Cd content, Se forms	2022.4.20	2022.5.12
CdSe3	50 $CdCl_2$+100 mg/L Na_2SeO_3	Root of CdSe3	Transcriptome, Phytohormone, Se content, Cd content, Se forms	2022.4.20	2022.5.12
		Shoot of CdSe3	Transcriptome, Physiological and biochemical indexes, Phytohormone, Se content, Cd content, Se forms	2022.4.20	2022.5.12

Table S2. Changes in the occurrence of cadmium in soil of mustard treated with different concentrations of Se and Cd.

Treatments	Se content (mg/kg)	The content of different species of Cd in the soil (mg/kg)						
		Water-soluble	Ion exchange	Humic acid bound	Iron manganese oxidation	Carbonate bound	Strong organic acid bound	Residual
CK	-	0.014	0.025	-	-	-	-	-
Cd2	-	6.327	13.139	3.458	3.622	1.867	0.218	0.334
CdSe1	4.108	6.151	12.078	3.723	2.359	1.790	0.192	0.145
CdSe2	22.533	5.698	11.927	3.332	2.587	2.131	0.390	0.319
CdSe3	43.671	5.905	11.485	3.815	3.053	2.073	0.165	0.157

Note: The horizontal line indicated that the substance was not detected in the sample. The lower detection limit of the method was Cd not less than 0.02, and Se not less than 0.01mg/kg.

Table S3. Annotation analysis of the first 30 hot spot genes screened in the Darkred module.

ID	PFAM description	BP Description	MF Description	CC Description
Cluster-6475.15625	Ankyrin repeat/Rotavirus VP3 protein	viral process	GTP binding	viral nucleocapsid
Cluster-6475.15747	--	--	--	--
Cluster-6475.16421	Protein tyrosine and serine/threonine kinase/BDHCT domain//Procyclic acidic repetitive protein (PARP)	DNA replication//protein phosphorylation	ATP binding//hydrolase activity, acting on acid anhydrides, in phosphorus-containing anhydrides binding//protein kinase activity	nucleus//membrane
Cluster-6475.17354	Ribosomal protein S21//Oxaloacetate decarboxylase, gamma chain//Conotoxin	proline metabolic process//translation//sodium ion transport//pathogenesis//ribosome biogenesis//arginine metabolic process//pyruvate metabolic process//sodium ion export across plasma membrane//transport	ion channel inhibitor activity//sodium ion transmembrane transporter activity//structural constituent of ribosome//oxaloacetate decarboxylase activity	membrane/extracellula r region/ribosome
Cluster-6475.17527	Flavin containing amine oxidoreductase//Pyridine nucleotide-disulfide oxidoreductase//FAD dependent oxidoreductase//Prenylcysteine lyase	oxidation-reduction process//prenylcysteine catabolic process	oxidoreductase activity, acting on a sulfur group of donors, oxygen as acceptor//oxidoreductase activity	--
Cluster-6475.18119	Ribosomal protein L4/L1 family	translation//ribosome biogenesis	structural constituent of ribosome	ribosome
Cluster-6475.19164	--	--	--	--
Cluster-6475.19754	Ribosomal L18 of archaea, bacteria, mitoch. and chloroplast	translation//ribosome biogenesis	structural constituent of ribosome	ribosome
Cluster-6475.21245	Photosystem II Pbs27//CHC2 zinc finger	transcription, DNA-templated//DNA replication, synthesis of RNA primer//DNA replication//photosystem II assembly	DNA primase activity//DNA binding//zinc ion binding	nucleolus//replication fork
Cluster-6475.21320	Ribosomal protein L13	translation//ribosome biogenesis	structural constituent of ribosome	ribosome
Cluster-6475.23405	Geminivirus putative movement protein//CG-1 domain	transport of virus in the host, cell to cell	DNA binding	An integral component of the membrane

Cluster-6475.23436	Ion transport protein//IQ calmodulin-binding motif	transmembrane transport//ion transport	protein binding//ion channel activity	membrane

Table S4. Annotation analysis of the first 30 hot spot genes screened in the Black module.

ID	PFAM description	BP Description	MF Description	CC Description
Cluster-6475.10477	Class-II DAHP synthetase family	tryptophan biosynthetic process//L-phenylalanine biosynthetic process//tyrosine biosynthetic process//aromatic amino acid family biosynthetic process	3-deoxy-7-phosphoheptulonate synthase activity	--
Cluster-6475.14765	--	--	--	--
Cluster-6475.16007	Legume lectin domain	--	carbohydrate binding	--
Cluster-6475.21817	Plant invertase/pectin methylesterase inhibitor//Syntaxin 6, N-terminal//Pectinesterase//Ubiquitin carboxyl-terminal hydrolase	cell wall modification//protein deubiquitination//starch metabolic process//sucrose metabolic process//Golgi vesicle transport	pectinesterase activity//enzyme inhibitor activity//thiol-dependent ubiquitinyl hydrolase activity	membrane
Cluster-6475.22115	--	--	--	--
Cluster-6475.22125	Calreticulin family	protein folding	unfolded protein binding//calcium ion binding	endoplasmic reticulum
Cluster-6475.23760	Ubiquinol cytochrome reductase transmembrane region//Rieske [2Fe-2S] domain	oxidative phosphorylation//oxidation-reduction process//obsolete electron transport	two iron, two sulfur clusters binding//oxidoreductase activity// ubiquinol-cytochrome-c reductase activity	--
Cluster-6475.24516	Phosphatidylinositol-4-phosphate 5-Kinase//Glutamine synthetase, beta-Grasp domain//Glutamine synthetase, catalytic domain	glutamine biosynthetic process//nitrogen compound metabolic process// phosphatidylinositol metabolic process// peptidoglycan biosynthetic process	glutamate-ammonia ligase activity//phosphatidylinositol phosphate kinase activity	--
Cluster-6475.25777	AP2 domain	regulation of transcription, DNA-templated	DNA-binding transcription factor activity	transcription regulator complex
Cluster-6475.28795	Aflatoxin regulatory protein	regulation of transcription, DNA-templated//aflatoxin biosynthetic process	DNA binding	nucleus
Cluster-6475.29255	Aminotransferase class I and II//Beta-eliminating lyase	cellular amino acid metabolic process//biosynthetic process	lyase activity//pyridoxal phosphate binding	--

Cluster-6475.29505	S-adenosylmethionine synthetase, C-terminal domain//S-adenosylmethionine synthetase, N-terminal domain//S-adenosylmethionine synthetase, central domain	S-adenosylmethionine biosynthetic process//methionine metabolic process	methionine adenosyltransferase activity	--
Cluster-6475.5177	Peptidase family M20/M25/M40	--	hydrolase activity	--
Cluster-6475.9444	--	--	--	--
Cluster-6475.12379	Pyridine nucleotide-disulfide oxidoreductase//Malic enzyme, N-terminal domain//Malic enzyme, NAD binding domain	pyruvate metabolic process//oxidation-reduction process//carbon utilization//tricarboxylic acid cycle//malate metabolic process	malate dehydrogenase (decarboxylating) (NAD+) activity//oxidoreductase activity//NAD binding	--
Cluster-6475.15282	Peroxidase	obsolete peroxidase reaction//response to oxidative stress//oxidation-reduction process	heme binding//peroxidase activity	--
Cluster-6475.16164	Saccharopine dehydrogenase NADP binding domain//3-beta hydroxysteroid dehydrogenase/isomerase family//NAD dependent epimerase/dehydratase family	androgen metabolic process//oxidation-reduction process//C21-steroid hormone metabolic process//steroid biosynthetic process//estrogen metabolic process	oxidoreductase activity//3-beta-hydroxy-delta5-steroid dehydrogenase activity//obsolete coenzyme binding//oxidoreductase activity, acting on the CH-OH group of donors, NAD or NADP as acceptor//catalytic activity	--
Cluster-6475.17754	PLAT/LH2 domain//Lipoxygenase	oxidation-reduction process	metal ion binding//protein binding//oxidoreductase activity, acting on single donors with incorporation of molecular oxygen, incorporation of two atoms of oxygen	--
Cluster-6475.18788	Small, acid-soluble spore proteins, alpha/beta type//BCCT, betaine/carnitine/choline family transporter//AMP-binding enzyme	DNA topological change//nitrogen compound transport	transmembrane transporter activity//catalytic activity//double-stranded DNA binding	membrane
Cluster-6475.20475	Divalent cation transporter	cation transport	cation transmembrane transporter activity	--
Cluster-6475.18032	Protein NRT1/ PTR FAMILY 8.1	transmembrane transport	transmembrane transporter activity	membrane
Cluster-6475.27175	Annexin	--	calcium ion binding//calcium-dependent phospholipid binding	--

Table S5. qRT-PCR validation primer for DEGs hot genes related to Se and Cd treatments.

Gene name	Primer1	Primer2
Actin	GGAGTGATGGTTGGAATGGGA	CCAAATCTTCTCCATGTCATCC
4CL1	CACGTGTACACTTACTCCGAC	GGAAAGAGAGGACGAACTCG
ABCC1	AGGAACCTCCCAAACATAACC	GGCTTGTTTGTGTCCTCTTG
DCT1	CGTCTCGCTCTCAATCAAGC	GAGCTTCTCTTTGGTGATCTC
DHAPS1	AGATCCCAAGAATGAATCCAT	AGTCCAGCTCCTTCTGATCC
NAD-ME2	TCCACGATCCATGGTTTAACA	CGAAAGGACTCAATGAAACG
NRT1	AGATAGCATCAAAGACGGTGA	CACTGTGGCAAAGACTATCC
PLAT	TAGCAAAGAGCCTCAGCTCT	CCGTGACCTTACATCGTCCA
PME3	CGTAGCCTTGCTACTCGTAA	GATGTTAACACTGCGTGAGA
UCCR1	GGTCCTTACTCAAGGAAACGA	AGAGGACGAAATAGGCGAAG
ZIPT4	CCATACCTCTCGTTGGCAA	CCAGCAAGCATGTGGACAA

Note: Using *B. juncea* Actin gene as internal reference gene.

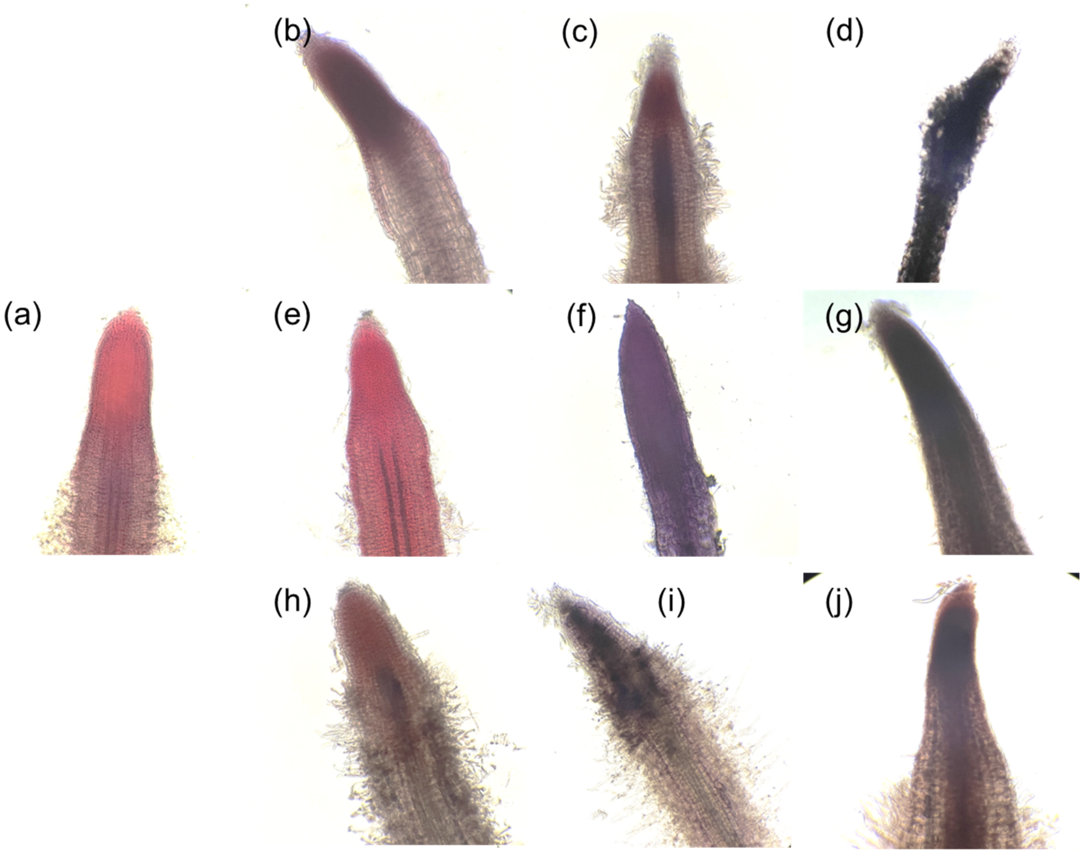

Figure S1. Effects of different concentrations of Se and Cd on the root tip growth of *B. juncea.* (a). CK; (b). Cd1, treatment of seedling root tips with 10 mg/L $CdCl_2$; (c). Cd2, 50 mg/L $CdCl_2$; (d). Cd3, 100 mg/L $CdCl_2$; (e). Se1 treatment of seedling root tips with 10 mg/L Na_2SeO_3; (f). Se2, 50 mg/L Na_2SeO_3; (g). Se3, 100 mg/L Na_2SeO_3; (h). CdSe1, 50 mg/L $CdCl_2$ and 10 mg/L Na_2SeO_3; (i). CdSe2, 50 mg/L $CdCl_2$ and 50 mg/L Na_2SeO_3; (j). CdSe3, 50 mg/L $CdCl_2$ and 100 mg/L Na_2SeO_3.

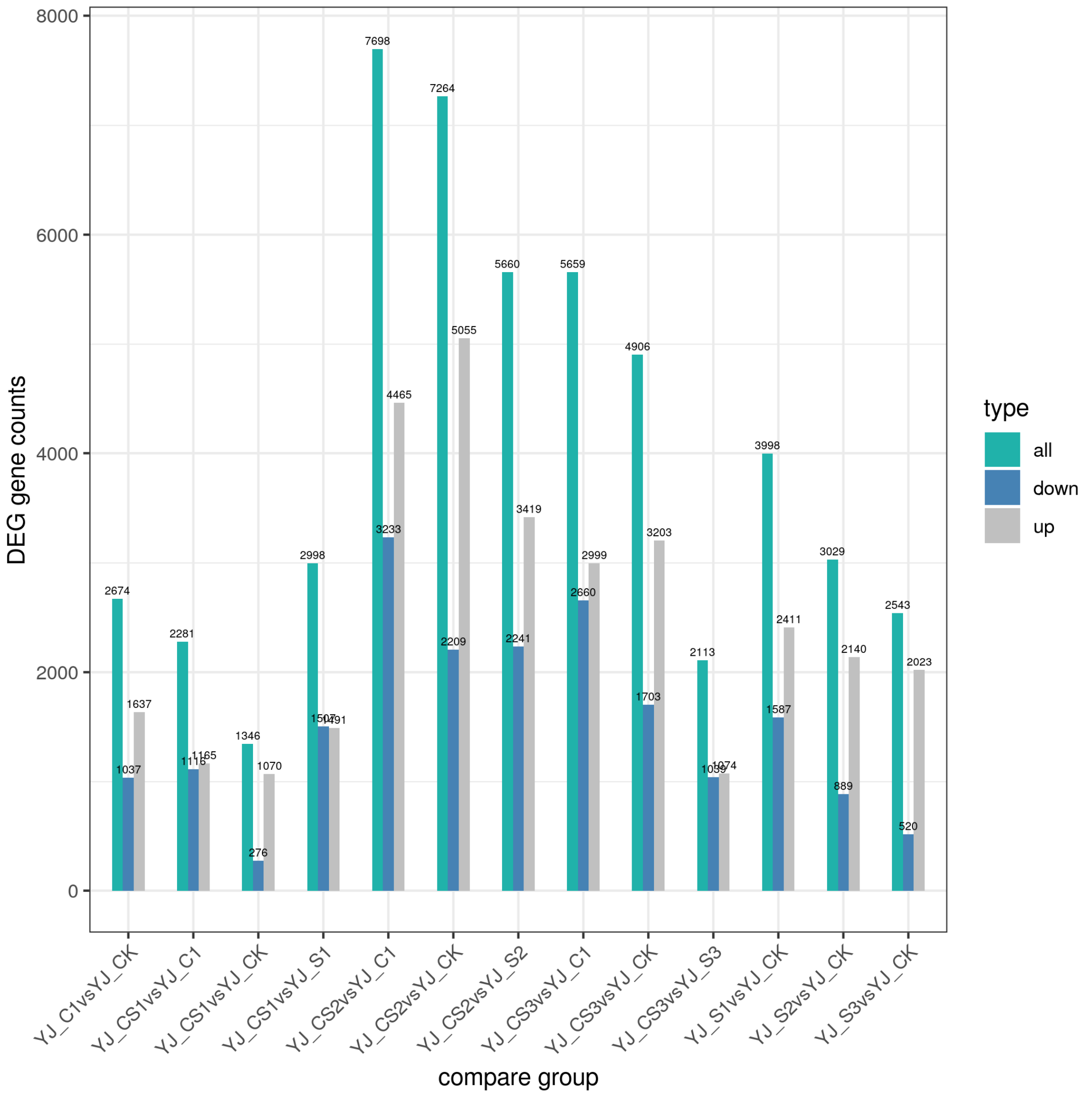

Figure S2. The number of DEGs among samples treated with different concentrations of $CdCl_2$ and Na_2SeO_3. The green histogram shows the total DEGs, the gray represents the number of upregulated DEGs, and the blue represents the number of downregulated DEGs.

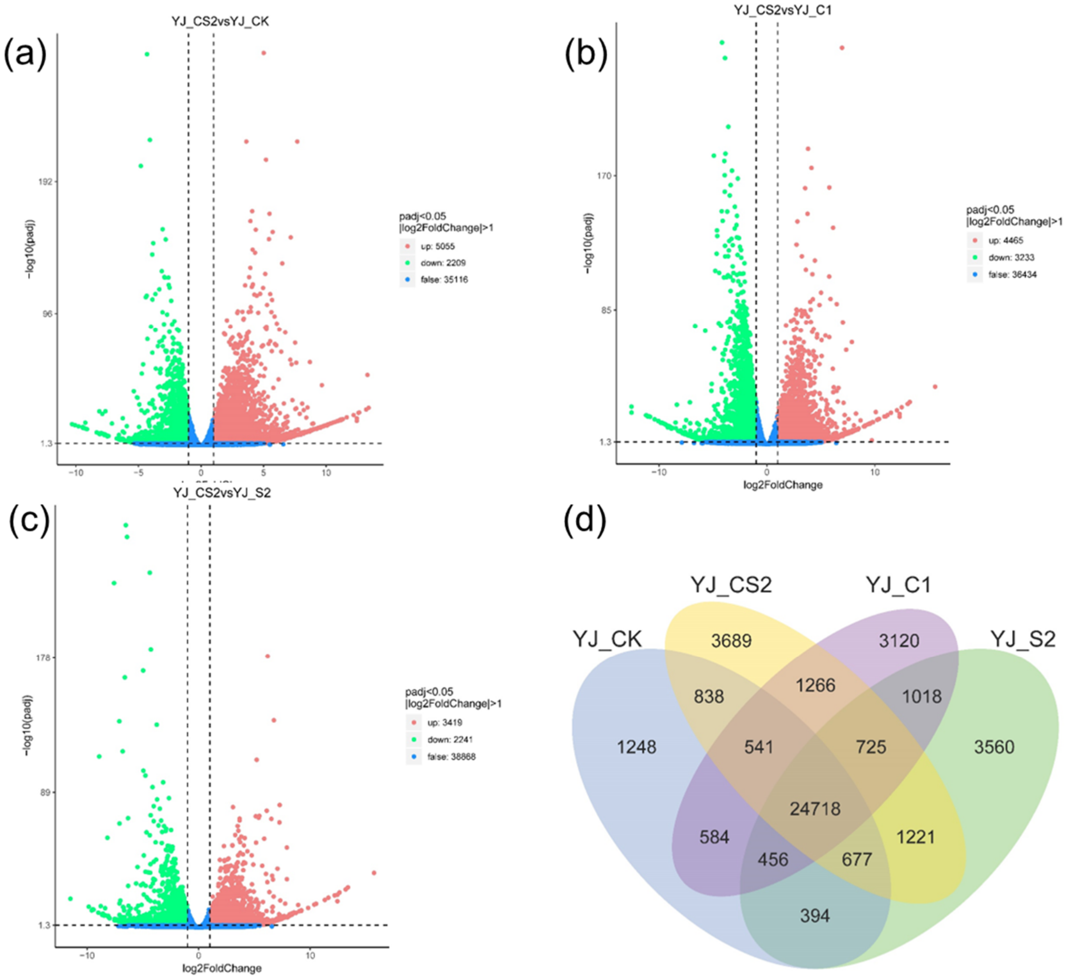

Figure S3. Volcano map and Venn diagram for the comparison of differentially expressed genes between samples treated with different concentrations of sodium selenite and cadmium chloride. (a) volcano map of DEGs between CdSe2 and control; (b) volcano map of DEGs between CdSe2 and Cd1; (c) volcano map of DEGs between CdSe2 and Se2 treatments and (d) Venn diagram of DEGs between CdSe2, Cd1, Se2 and control group.

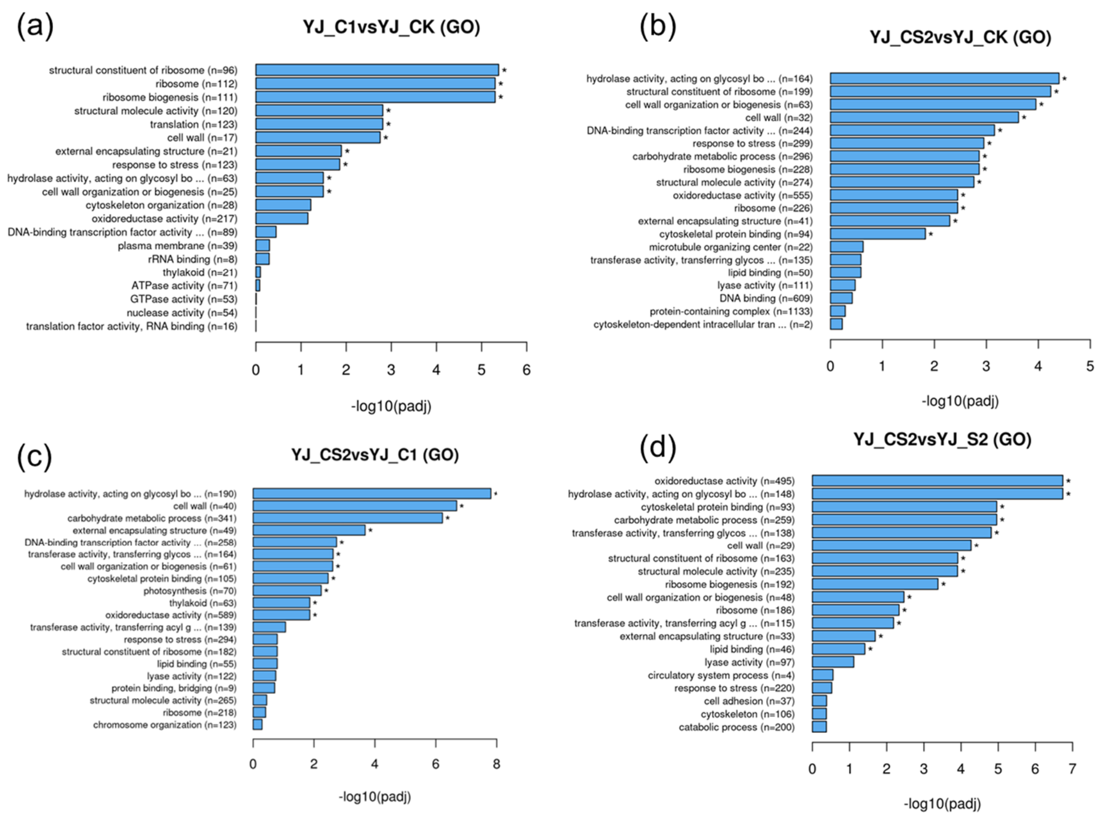

Figure S4. GO enrichment analysis of DEGs in samples treated with different concentrations of $CdCl_2$ and Na_2SeO_3. (a) GO enrichment of DEGs between Cd1 and control group; (b) GO enrichment of DEGs between CdSe2 and control group; (c) GO enrichment of DEGs between CdSe2 and Cd1 and (d) GO enrichment of DEGs between CdSe2 and Se2 treatments.

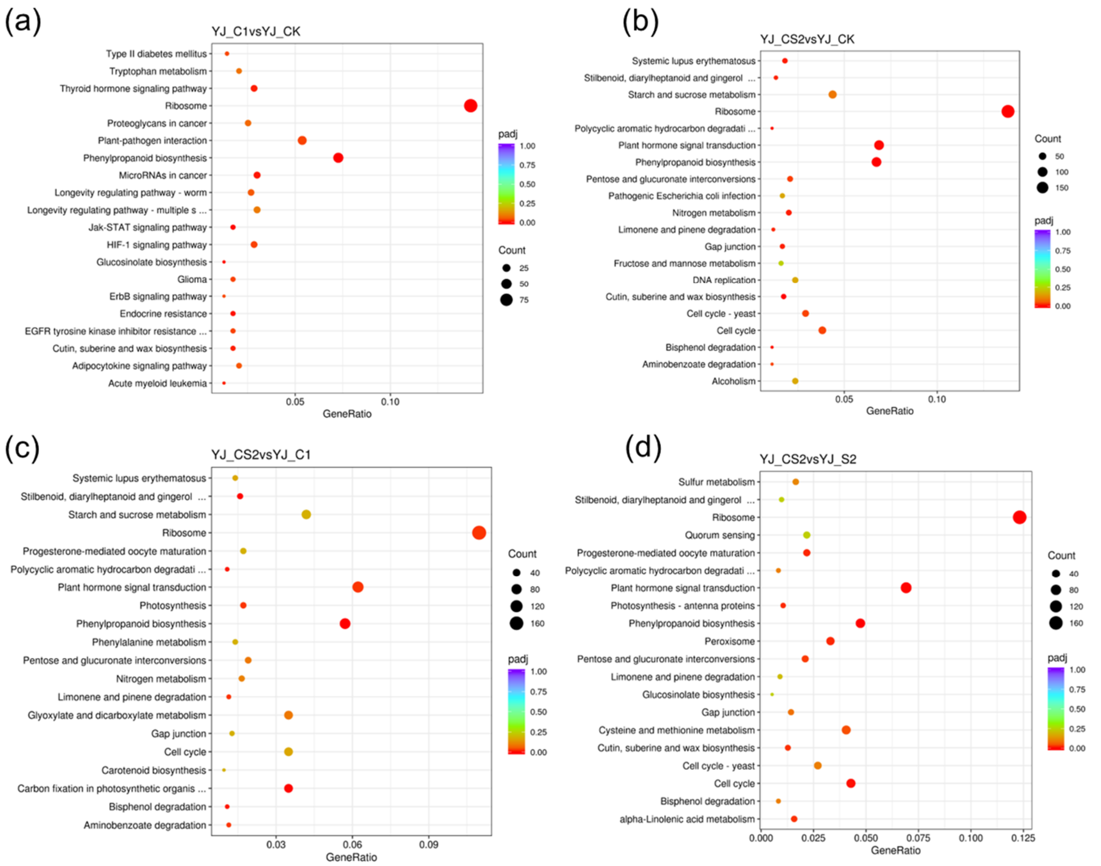

Figure S5. KEGG pathway enrichment analysis of DEGs in samples treated with different concentrations of $CdCl_2$ and Na_2SeO_3. (a) KEGG pathway enrichment of DEGs between Cd1 and control group; (b) KEGG pathway of DEGs between CdSe2 and control group; (c) KEGG pathway of DEGs between CdSe2 and Cd1; and (d) KEGG pathway of DEGs between CdSe2 and Se2 treatments.

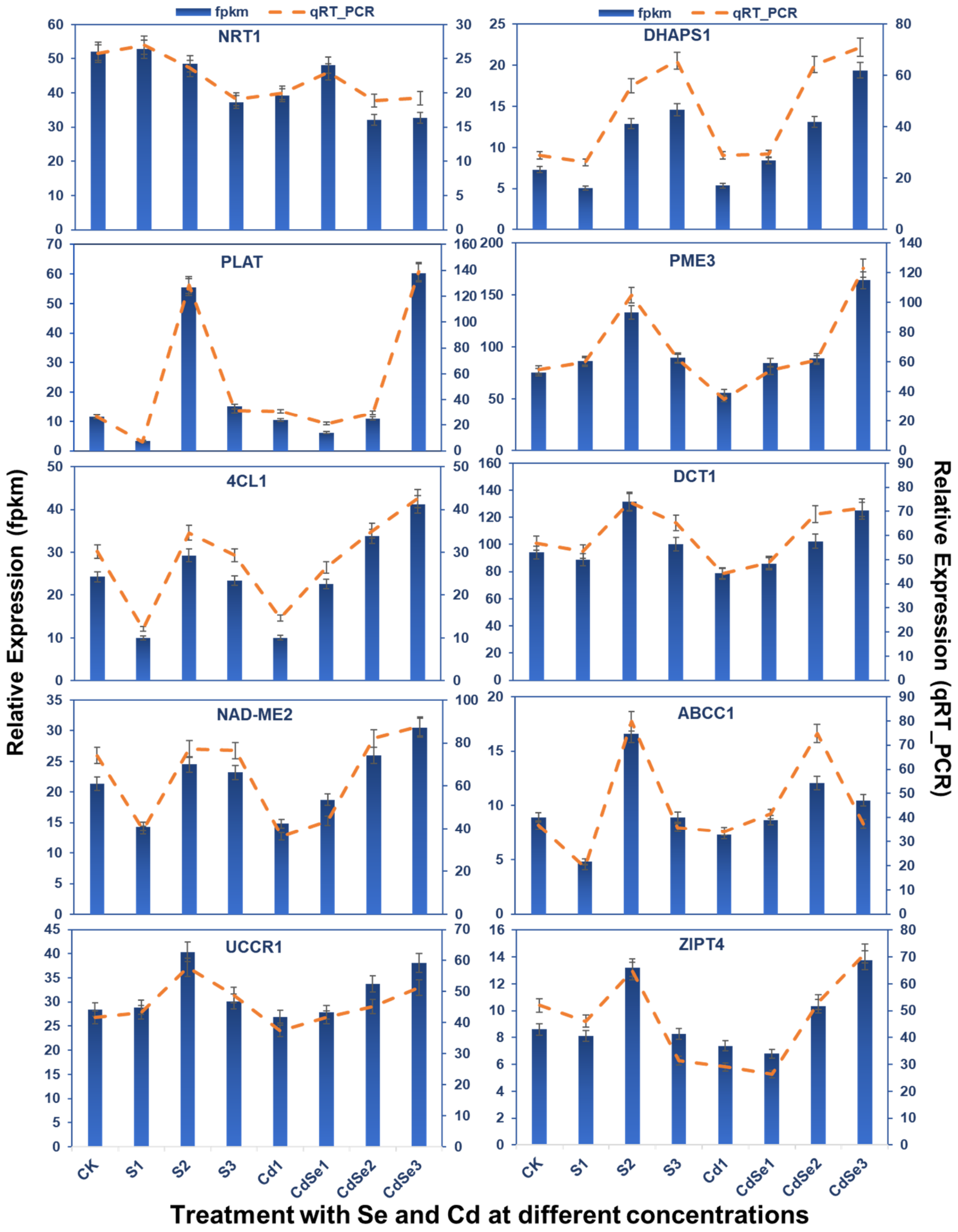

Figure S6. Comparative validation of qRT-PCR and RNA-seq of candidate genes related to Se and Cd uptake, transport and metabolism in *Brassica juncea*. The *Actin* gene was used as control, and the $2(^{-\Delta\Delta Ct})$ analysis method was used to determine the relative expression levels of genes.

The error bars represent the SD of three biological replicates. The numbers above the graphics correspond to values obtained with the correlation analysis of the gene expression ratios obtained from the RNA-seq data (blue column) and the qRT-PCR data (yellow line). CK: control samples; S1 is a mustard sample treated with 10 mg/L Na_2SeO_3; S2 is a mustard sample treated with 50 mg/L Na_2SeO_3; S3 is mustard sample treated with 100 mg/L Na_2SeO_3; Cd is a mustard sample treated with 50 mg/L $CdCl_2$; CdSe1 is the mustard sample treated with 50 mg/L $CdCl_2$+10 mg/L Na_2SeO_3; CdSe2 is the mustard sample treated with 50 mg/L $CdCl_2$+50 mg/L Na_2SeO_3; and CdSe3 is the mustard sample treated with 50 mg/L $CdCl_2$+100 mg/L Na_2SeO_3.
